# A new NMVOC speciated inventory for a reactivity-based approach to support ozone control strategies in Spain

**DOI:** 10.1016/j.scitotenv.2023.161449

**Published:** 2023-04-01

**Authors:** K. Oliveira, M. Guevara, O. Jorba, X. Querol, C. Pérez García-Pando

**Affiliations:** a Barcelona Supercomputing Center, Barcelona, Spain; b Institute of Environmental Assessment and Water Research (IDAEA), Spanish Research Council (CSIC), Barcelona, Spain; c ICREA, Catalan Institution for Research and Advanced Studies, Barcelona 08010, Spain

**Keywords:** NMVOC speciation, Anthropogenic emissions, Emission control strategies, OFP, Tropospheric ozone

## Abstract

Ozone (O_3_) pollution is a persistent problem in many regions of Spain, so understanding O_3_ precursor emissions and trends is essential to design effective control strategies. We estimated the impact of Non-Methane Volatile Organic Compounds (NMVOC) species upon O_3_ formation potential (OFP) using the maximum incremental reactivity approach. For this, we developed a speciated NMVOC emission inventory for Spain from 2010 to 2019 combining national reported emissions with state-of-the-art speciation profiles, which resulted in a database of emissions for over 900 individual NMVOC species and 153 individual sectors. Additionally, we analysed 2030 emission projections to quantify the expected impact of planned measures on future OFP levels. Overall, the main activities contributing to OFP in Spain are paint manufacturing and applications (20 %), manure management (16 %), and domestic solvent use (6 %). These activities contribute unevenly across regions. The more urbanised areas report a larger contribution from the solvent sector (64 % in Madrid), while in rural areas, manure management and agricultural waste burning gain importance (24 % in Extremadura), indicating that local control measures should be implemented. The top 10 NMVOC species contributing to OFP are ethanol, ethene, xylenes, propene, toluene, formaldehyde, 1,3-butadiene, styrene, n-butane, and cyclopentane, which together are responsible for 54 % of the total OFP. Our trend analysis indicates a reduction of NMVOC emissions and OFP of −5 % and −10 % between 2010 and 2019, respectively. The larger decrease in OFP is driven by a bigger reduction in xylenes (−29 %) and toluene (−28 %) from paint application industries and the road transport sector. By 2030 a significant increase (+37 %) in the OFP from the public electricity sector is expected due to the planned increase in biomass use for power generation. Our results indicate that policies should focus on paint reformulation, limiting aerosol products, and implementing NMVOC control devices in future biomass power plants.

## Highlights

• A new speciated anthropogenic NMVOC emission inventory was developed for Spain.
• State-of-the-art NMVOC speciation profiles were compiled and compared.
• Ozone formation potential (OFP) was quantified through a reactivity approach.
• Main activities and species contributing to OFP were identified per region.
• A set of policy recommendations was proposed for mitigation strategies.

## Graphical abstract

**Figure:**
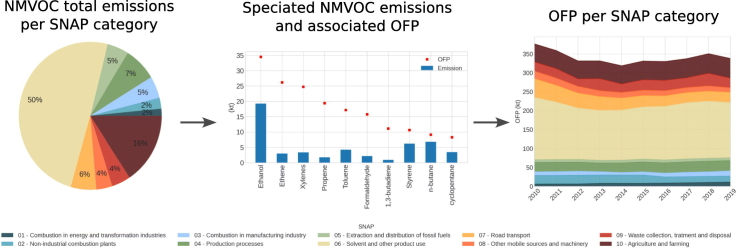

## 1 Introduction

Tropospheric ozone (O_3_) affects human health (WHO, 2021), biodiversity (Agathokleous et al., 2020; Mills et al., 2013), vegetation (Braun et al., 2017; Mills et al., 2013, Mills et al., 2018), crops (Schucht et al., 2021; Tai et al., 2014; Van Dingenen et al., 2009), and infrastructures (Kumar and Imam, 2013). In Spain, several efforts have been made to regulate and reduce air pollution during the last decades (MITECO, 2021). However, despite the overall reduction trends in precursor emissions, most of the O_3_ indicators to assess the target values and long-term objectives set up by the European Ambient Air Quality Directive (EC, 2008) have shown very low decreasing trends and even increases in specific atmospheric basins between 2000 and 2019 (EEA, 2020; Massagué et al., 2022; Querol et al., 2016). Regarding the target value of O_3_ for health protection, 34 out of the 127 evaluated areas in 2019 show values above the target value, 81 show values between the target value and the long-term target value, and the remaining 12 are below the long-term objective. These results have not substantially improved since 2011 and have been constant since 2016 (MITECO, 2019a). Spain is even farther away from the O_3_ target values (Bowdalo et al., 2022) recommended by the recently updated WHO guidelines (WHO, 2021).

Non-Methane Volatile Organic Compounds (NMVOC) play a major role in the formation of O_3_ (Monks et al., 2015) and are emitted from two major sources, i.e., anthropogenic volatile organic compounds (AVOC) emitted from human activity and biogenic volatile organic compounds (BVOC) from terrestrial ecosystems (Guenther et al., 1995). In Spain, anthropogenic NMVOC emissions diminished between 2010 and 2014 (−10 %) and increased thereafter to the point that in 2019, emissions were only −1.4 % lower than in 2010. In 2019, the solvent sector was the primary sector contributing to NMVOC (42 %), followed by agriculture (14 %), livestock (13 %) and industry (10 %) (MITERD, 2021a). According to the official Spanish emission projections (MITERD, 2021b), NMVOC emissions are expected to be reduced by 11 % in 2030 relative to 2019 in the scenario with additional measures (WaM). This overall reduction results from decreases in the residential, commercial and institutional sector (−52 %), agricultural soils (−22 %), waste management (−61 %), and the solvent sector (−5 %); and increases in the road transport sector (+15 %), mainly due to a rise in the share of gasoline vehicles, and in the public energy sector (+26 %), mainly due to a 77 % increase in biomass usage to produce electricity (IEA, 2021).

NMVOC can be oxidised by hydroxyl radicals (·OH) in the presence of sunlight, yielding oxygenated VOC (OVOC), organic radicals (RO_2_) and hydro-peroxyl radicals (HO_2_). These two radicals oxidise nitrogen oxide (NO) into nitrogen dioxide (NO_2_). The later photo-dissociates into NO and O* (activated oxygen), which reacts with oxygen (O_2_) to generate O_3_ (EPA, 2006b). O_3_ formation potential (OFP) is a parameter that has been widely used to rank and describe the role of individual NMVOC in O_3_ formation, which is incorporated into the policy-making process to develop more cost-effective control strategies (Capps et al., 2010; Hakami et al., 2004). The OFP of each NMVOC is quantified by its maximum incremental reactivity (MIR), defined as the number of additional grams of O_3_ formed per gram of NMVOC compound added to a representative atmospheric system (Carter, 2010). Many works, mainly in Asia (e.g. Fu et al. (2020); Li et al. (2019); Liang et al. (2020); Wang et al. (2018)), have used this reactivity-based approach to design strategies for tackling O_3_ problems, showing more effectiveness than the traditional mass-based approach (Derwent et al., 2007; Ou et al., 2015).

The reactivity-based approach requires as input information emissions of individual NMVOC species. However, this information cannot be obtained from official emission inventories reported under the Convention on Long-Range Transboundary Air Pollution (CLRTAP) or the Directive (EU) 2016/2284 on the reduction of national emissions of certain atmospheric pollutants, where NMVOC are only required to be provided as totals. To overcome this limitation, during the last years, non-official and science-based emission inventories have been developed in Europe to provide information on speciated NMVOC, including the Emissions Database for Global Atmospheric Research (EDGARv4.3.2) (Huang et al., 2017) and the CAMS regional inventory (CAMS-REG) (Kuenen et al., 2022). Despite being two well-recognised inventories, these two databases cannot be used for OFP estimation since they provide NMVOC emissions grouped into 25 species groups as proposed within the Global Emission Inventory Activity (GEIA), and the estimation of the OFP requires information from individual species. Therefore, the construction of a Spanish speciated NMVOC inventory is required to estimate OFP in Spain. In order to disaggregate total NMVOC by species, one common approach is to use chemical speciation profiles. Some examples of the most commonly used speciation profile databases are the SPECIATE database from the United States Environmental Protection Agency (US EPA) (Simon et al., 2010), the California Air Resources Board (CARB) database (CARB, 2022), the Institute of Energy Economics and Rational Energy Use (IER) database (Theloke and Friedrich, 2007), and Passant (2002).

Our work identifies the main species and sources contributing to OFP in Spain. To achieve this, we developed a speciated NMVOC inventory for anthropogenic emissions between the years 2010 to 2019, which is used to estimate the OFP for each individual source and species. For the main NMVOC contributing sources, we compiled and compared traditional chemical speciation profile databases with more recent literature and assessed their sensitivity to OFP calculation. Moreover, to assess the impact of future planned emission abatement strategies, we estimated the expected OFP in 2030, taking into account official emission projections reported under the CLRTAP. Based on our results, we propose a set of effective recommendations to reduce emissions from the main NMVOC reactive species in Spain.

Section 2 describes the development of the NMVOC speciated inventory along with the estimation of the OFP for each anthropogenic source. Section 3 shows the results of the speciated NMVOC emission inventory and a comparison with CAMS-REG speciated emission inventory, an OFP sensitivity analysis, and discusses the OFP results at national and autonomous community (NUTS 2) levels. This section also evaluates the impact of 2030 projections on OFP. Section 4 presents our main conclusions, a set of recommendations to reduce NMVOC emissions, the limitations and uncertainties when using this approach, and finally, planned future work.

## 2 Methodology

### 2.1 Speciated NMVOC emission inventory

We estimated speciated NMVOC emissions based on the 2010–2019 Spanish officially reported NMVOC emissions to the CLRTAP and European Union (EU) Directive 2016/2284, prepared by the Ministry for the Ecological Transition and the Demographic Challenge (MITERD). Total NMVOC emissions following the Selected Nomenclature for reporting of Air Pollutants (SNAP) classification system were split into individual chemical species following a profile-assignment approach. The Nomenclature For Reporting (NFR) system is the current official format to report emission inventories under the CLRTAP. Additionally, the MITERD also provides the emissions using the SNAP classification system. Overall, since the SNAP system provides a higher level of detail for the speciation of individual pollutant activities, this was selected over the NFR system. For instance, SNAP includes 9 different activities (SNAP 060101 to 060109) for paint applications, while NFR only has 1 category (i.e., Coating applications, 2D3d). Similarly, for emissions related to the use of solvents in the manufacturing and processing of chemical products, the SNAP nomenclature includes 14 different activities (SNAP 060301 to 060314), whereas, in NFR, all emissions are reported under the “Chemical products” category (2D3g). Finally, emissions from processing activities in the chemical industry are reported in 37 separate activities in SNAP (SNAP 0404 and 0405), while in NFR, they are all aggregated in the “Chemical industry” category (2B10a). For the same reason, the official gridded reported emissions at 0.1 × 0.1 degrees provided by parties to the LRTAP Convention could not be used in this work to derive gridded speciated NMVOC emissions, as they are provided following the Gridded NFR (GNFR) nomenclature, which consists of only 14 aggregated sector. The description of each SNAP activity and its mapping to the corresponding NFR category can be found in Table S1 of the Supplementary material.

To develop a speciated NMVOC inventory for Spain, a total of 153 SNAP categories related to national anthropogenic emissions (i.e., SNAP 01 to 10) were accounted for, excluding SNAPS 100102 and 100105, which relate to biogenic emissions derived from crops, and SNAP 080402, 080403, 080404, 080502, 080503, and 080504, which are associated to shipping, and aviation emissions above 1000 m. Biogenic emissions from terrestrial ecosystems (SNAP 11) were excluded mainly because this study focuses on sources that can be regulated by public authorities. Despite their significant contribution to total Spanish NMVOC (approximately 70 % according to Pay et al., 2019), depending on the NMVOC/NOx regime (Pun et al., 2002) and the interactions with anthropogenic species, BVOC impact on O_3_ can be lower than the impact of anthropogenic emissions (Sartelet et al., 2012).

The source of the chemical speciation profiles used in this work is presented in the Supplementary material Table S1. Most of the selected profiles are from the US EPA SPECIATE 5.1 database (Bray et al., 2019; Simon et al., 2010), which contains over 2000 organic gas profiles, followed by Theloke and Friedrich (2007), containing 87 speciation profiles for Europe, and Passant (2002), including many sources for the United Kingdom. For the most important NMVOC sources, speciation profiles were derived from state-of-the-art European literature. This includes, for instance, domestic use of solvents (Heeley-Hill et al., 2021), exhaust emissions from mopeds (Adam et al., 2010) and passenger cars (Marques et al., 2022). We also considered profiles available from the NMVOC reports compiled by the MITERD and Spanish regional governments under the national Real Decreto 117/2003 (MPR, 2003) (transposed from the Directive 1999/13/EC (EC, 1999)). The reports cover information on emissions of individual NMVOC species released from industrial and commercial facilities between 2017 and 2019.

For several SNAP activities, the most representative speciation profile was selected, taking into account the information provided by the datasets mentioned above. The selection criteria considered: 1) location, where the measurements to derive the profile were taken, the preference being Spain or Europe; 2) year of reference of the profile, the preference being the most recent year; 3) technologies/products considered by the profile (e.g., EURO categories for road transport related profiles, types of products for the use of solvents related profiles), the preference being the profile that better represents current technologies and products; and 4) the quality of the profile, i.e. the fraction of unknown species and detail of higher‑carbon, along with the total amount of species measured. The information for these parameters of each profile per activity can be found in Table S2 of the Supplementary material.

One limitation of the SNAP nomenclature, shared by the NFR structure, is that it does not report emissions by fuel type. This limitation is very relevant when it comes to the speciation of NMVOC released from combustion sources, such as the energy industry (SNAP 01), residential and commercial combustion (SNAP 02), manufacturing industry (SNAP 03) and road transport (SNAP 07). For instance, the composition of NMVOC emissions from natural gas-fired power plants is very different from the ones released from coal-fired power plants (Li et al., 2019). To overcome this limitation, we implemented a weighted approach (WA), by which we split the original SNAP emissions among fuels considering specific sources of information. For example, NMVOC exhaust emissions from passenger cars were split among fuels (i.e., gasoline, diesel and LPG) using the information provided by the CAMS-REG-APv5.1 emission inventory for Spain (Kuenen et al., 2022). For industrial or residential NMVOC emissions, the WA considered annual energy consumption statistics provided by IDAE (IDAE, 2019).

The speciation profiles selected and the assumptions made for each SNAP activity can be found in Table S1 of the Supplementary material.

**Fig. 1:**
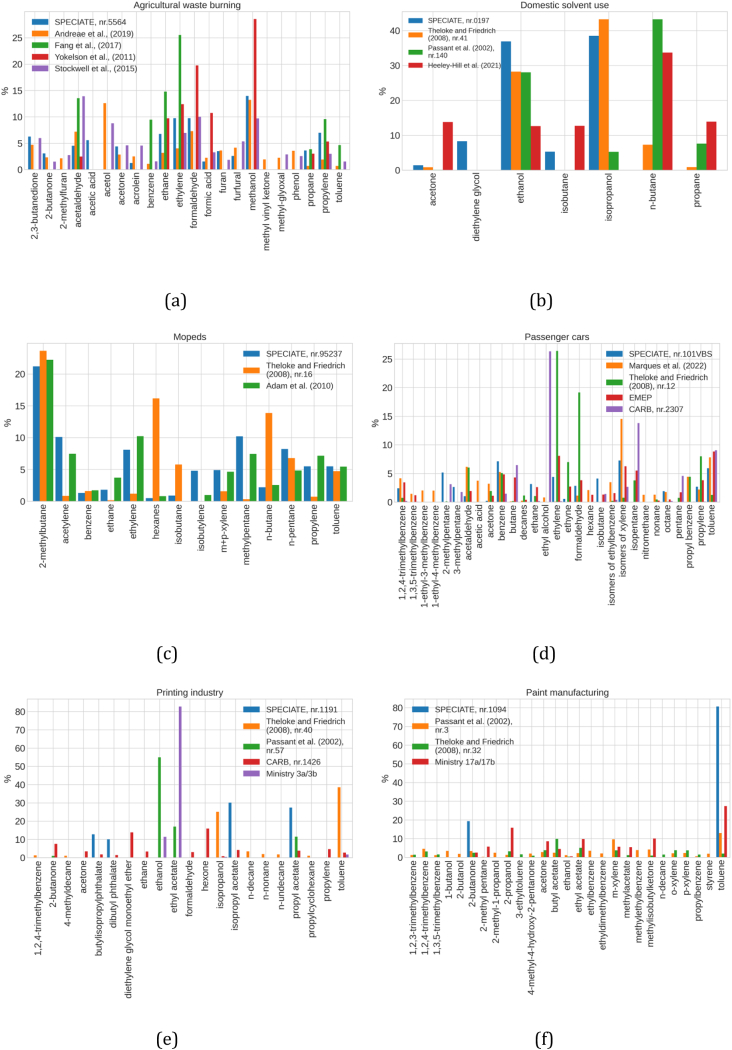
Speciation profile comparison for specific activities.

For the most relevant SNAP categories, which together contribute >65 % of the total NMVOC emissions in Spain, we collected and compared multiple speciation profiles to select the most appropriate one. For illustrative purposes, Fig. 1 presents the comparison for some of these sectors; the remaining sectors can be found in Fig. S1 of the Supplementary material. In order to facilitate the analysis and discussion of this comparison exercise, we only accounted for the major species of each speciation profile (i.e., species that sum up >60 % of the total NMVOC composition). Below we summarise the main differences between profiles for each source:

• For the agricultural waste burning sector, there is a large discrepancy among the NMVOC compositions reported by each of the selected profiles (Fig. 1a) (Andreae, 2019; Stockwell et al., 2015). The review work in Andreae (2019), which accounts for multiple measurements worldwide, showed that overall, methanol, formaldehyde, acetonitrile, acetaldehyde and ethylene are species commonly identified in the speciation profiles for this sector. For example, methanol, which is naturally found in vegetation, shows a significant variability for the multiple profiles presented, mainly due to differences in the vegetation type considered. Also, since methanol is widely used as an inert ingredient in agricultural and residential-use pesticides (EPA, 2006a), higher contribution levels could indicate crops/regions with more intense use of pesticides. For this sector, we used the profile from Stockwell et al. (2015) as it accounts for a wide variety of fuels and, therefore, could better represent this activity in Spain.
• The domestic solvent use (Fig. 1b) accounts for multiple products, including cosmetics and toiletries, car care products, household products, and DIY/buildings products (EMEP/EEA, 2019b). For each case, these can be aerosol or non-aerosol products, leading to large variability in the chemical composition of the associated NMVOC emissions. Aerosol products are the major sources of indoor air pollution (Nourian et al., 2021), mainly due to the high levels of harmful NMVOC that contain alcohol compounds, as well as propane, isobutane, and butane, which are common aerosol propellants and are found in many European household products (e.g., UNILEVER (2022)). The speciation profile from Passant (2002) only accounts for aerosol solvent-based domestic products, and therefore, it shows different dominating species (e.g. n-butane, 43 %) when compared to the profiles from SPECIATE (ref. number 0197) and Theloke and Friedrich (2007), which accounts only for a small percentage of products containing aerosol propellant and reports as dominant species isopropanol (39 %–43 %) and ethanol (28 %37 %). We selected the profile from Heeley-Hill et al. (2021), which is more recent (the other profiles are from before 1999), and it accounts for both aerosol and non-aerosol products, where n-butane (34 %) dominates, followed by propane (14 %) and acetone (14 %).
• For mopeds (Fig. 1c), all the profiles selected were measured in Europe and agree overall, with 2-methylbutane being the dominating species (ranging between 21 % and 23 %). The exception is Theloke and Friedrich (2007), which attributes a significant contribution to n-butane (14 %) and hexane (16 %). This could be explained by the different reference years of the profiles. The SPECIATE profile, based on the work of Montero et al. (2010) and the profile by Adam et al. (2010), are both from 2010, while Theloke and Friedrich (2007) reports data from 1992. Vehicle technologies have changed substantially between the 90s and the first decade of the 2000s, and this could play a significant role in the results. For this sector, we used the profile from Adam et al. (2010) because it accounts for multiple engine types of EURO-2 mopeds, while the profile from SPECIATE includes EURO-2 mopeds but also outdated technologies, i.e. pre-EURO and EURO-1 mopeds.
• As expected, passenger car profiles (Fig. 1d) include a large number of variables and assumptions and, therefore, distinct compound contributions. Further differences were expected as SPECIATE and CARB are based on USA vehicles and fuel, while EMEP, Theloke and Friedrich (2007) and Marques et al. (2022) measured emissions from European vehicles. For this sector, we used the most recent profile proposed by Marques et al. (2022) since it accounts for European fuel characteristics and also for recent EURO vehicles.
• For the printing industry (Fig. 1e), the reported NMVOC compositions differ substantially among selected profiles due to the dependency of NMVOC emissions upon ink type and printing technique (Saengchairat et al., 2017). For example, flexographic printing is a commonly used technique in labelling and packaging (Saengchairat et al., 2017) that typically uses water-based inks. In contrast, other techniques rely on the use of solvent-based inks (EMEP/EEA, 2019d), leading to different NMVOC speciation. Moreover, emissions of NMVOC from printing have been significantly impacted by the introduction of the so-called VOC Solvents Emissions Directive (i.e., Directive 1999/13/EC) approved in March 1999 (EC, 1999) and later amended through article 13 of the Paints Directive (i.e., Directive 2004/42/EC). All the profiles compiled for this sector are based on studies performed before the implementation of this directive (except for profile CARB nr.1426, for which no information is available), and subsequently, they may not represent the present day. Therefore, we selected the profile derived from the Spanish NMVOC reports compiled by the Spanish Ministry under the framework of the Real Decreto 117/2003, which reports ethyl acetate (83 %) and ethanol (11 %) as the dominant species.
• For emissions from the paint manufacturing sector (Fig. 1f), the year of the profile is key, given the transition from solvent-based to water-based paints during the last decade triggered by European Directives. Toluene, isopropanol, acetone and butyl acetate are typically the dominating NMVOC in solvent-based paint emissions, while xylene is usually detected in high concentrations in emissions linked to water-based paints (Bråtveit et al., 2004). This fact explains the discrepancy between the SPECIATE profile, which is based on data from 1989, and the other more recent profiles. For this sector, we considered the speciation profile derived from the Spanish NMVOC reports compiled under the Real Decreto 117/2003.

Once all SNAP categories were assigned with a specific chemical profile, the emissions of each individual NMVOC species were estimated according to Eq. (1).

(1) $$E_{i}=\sum_{j}E_{j}\times R_{\mathrm{ij}}$$

where:

*i* = specific NMVOC specie
*j* = SNAP activity
E_*i*_ = total emission of the specie *i*
R_*ij*_ = ratio of specie *i* from SNAP source *j*

The resulting speciated NMVOC emission inventory accounts for a total of over 900 individual species.

### 2.2 Estimation of ozone formation potential (OFP)

To evaluate the effects of individual NMVOC species and emission sources on O_3_ formation, we applied the widely used maximum incremental reactivity (MIR) (Carter, 1994) method. The MIR values were adopted from Carter, 1999, Carter, 2009, Carter, 2010 with the recent updates from Venecek et al. (2018). The MIR values for each modelled compound were obtained by applying a chemical mechanism in a box model to calculate organic reactivities for chemical conditions observed in cities in the United States (Venecek et al., 2018).

Similarly to the speciated emission inventory by mass described in Section 2.1, an OFP-based NMVOC emissions inventory was constructed following Eq. (2):

(2) $${\mathrm{OFP}}_{i}=\sum_{j}E_{\mathrm{ij}}\times {\mathrm{MIR}}_{i}$$

where:

*OFP*_*i*_ = total O_3_ formation potential of species *i*
*E*_*ij*_ = emission of the species *i* for SNAP source *j*
*MIR*_*i*_ = maximum increment reactivity of species *i*

Due to a lack of MIR for certain species or species classified as unknown compounds, 0.8 % of the total emissions were not considered in the OFP quantification analysis.

The OFP was estimated for the period when O_3_ levels are at their maximum, i.e., between June and August (Querol et al., 2016). To do so, annual NMVOC emissions for each SNAP activity were temporally disaggregated using the sector-specific monthly profiles from Guevara et al. (2020). The profiles were obtained from multiple sources of information, mainly including reported activity data that can be linked to each specific sector, such as energy production statistics, traffic counts or industrial production indexes. For example, the temporal profiles related to agricultural waste burning activity show a high variability per source of information but also per country and per year. So, for this work, we estimated the average monthly variation for Spain between 2010 and 2019 provided by Global Fire Emissions Database (GFED) (van der Werf et al., 2017). Fig. S2 of the Supplementary material shows the monthly variation of sector contribution to total NMVOC emissions for Spain and specific regions in 2019. Although this study focuses on July and August, the temporal profiles showed that the NMVOC emissions have significant monthly variation, with sector contributions changing significantly between winter and summer (e.g. residential combustion (SNAP 02) is reduced during summer).

## 3 Results and discussion

### 3.1 Speciated NMVOC emissions and trends

**Fig. 2:**
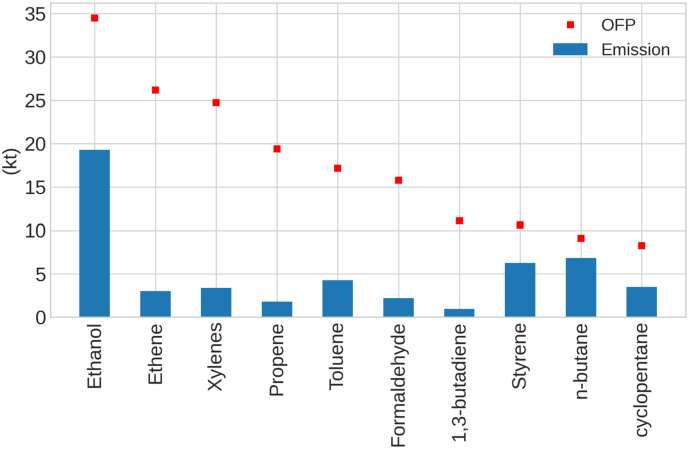
Comparison of emissions and OFP (kt) of the top ten OFP-based NMVOC species, between June and August, in 2019. Where “Xylenes” includes all isomers of xylene (o-xylene, m-xylene, and p-xylene).

Fig. 2 shows the emissions (kt) and OFP (kt) for the top ten species contributing to total OFP in 2019 (i.e., 54 % of the total OFP). According to the results, ethanol, ethene, isomers of xylene (i.e., o-xylene, m-xylene, and p-xylene), propene, toluene, formaldehyde, 1,3-butadiene, styrene, n-butane and cyclopentane are the top 10 species in terms of OFP. Ethanol is the major emitted species (19 kt in 2019) and contributor to total OFP (35 kt, 11 % of the total OFP), followed by ethene (26 kt, 8 % of the total OFP) and isomers of xylene (24 kt, 7 % of the total OFP). Despite presenting larger emissions than other species, styrene and n-butane present a lower contribution to total OFP (3.2 % and 2.7 %, respectively). The different patterns between emissions and OFP are related to the difference in the reactivity between species, e.g., ethene presents a MIR value of 8.6, which is almost 7 times higher than the one for n-butane (1.3).

**Fig. 3:**
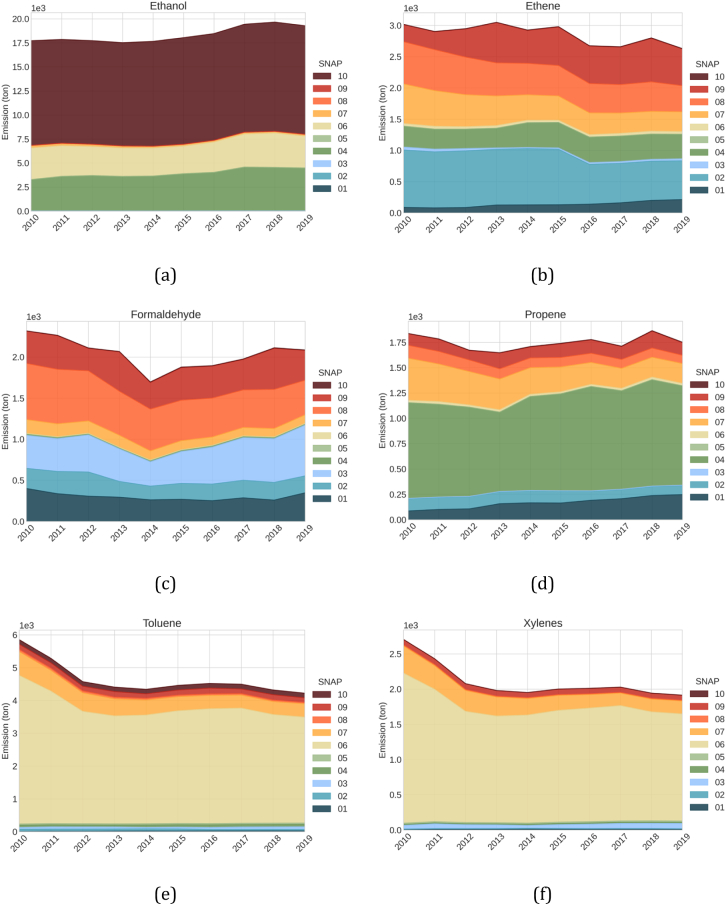
Emission trends of the top 6 NMVOC species (t) contributing to OFP by SNAP sector between 2010 and 2019 (June to August).

Fig. 3 shows the emission trends of the top 6 NMVOC species (kt) contributing to OFP by SNAP sector during 2010–2019.

According to results, ethanol has been slightly increasing since 2014, mainly due to an increase in emissions from industrial processes (SNAP 04), namely the food and beverages industry, such as the manufacturing of bread (SNAP 040605) and spirits (SNAP 040608). Livestock and their waste are responsible for around 60 % of ethanol emissions, followed by bread production (12 %) and domestic solvent use (9 %), where ethanol is a common ingredient in many cosmetics and beauty products.

Ethene (or ethylene) emissions were quite constant after a significant drop in residential combustion emissions in 2016 (−29 % when compared to 2015). Approximately 22 % of ethene emissions come from residential combustion (SNAP 020202) and 13 % from industrial waste incineration (SNAP 090202), with the first gaining even more importance when looking at the whole year. The increase in the contribution from SNAP 09 is mainly caused by the increase in emissions from industrial waste incineration, in which ethene has a significant share (44 %). Because ethene is mainly produced during the combustion of biomass, gasoline and natural gas fuels, multiple emission sources significantly contribute to its total emissions (Morgott, 2015).

Between 2010 and 2019, formaldehyde emissions reached a minimum in 2014 and have been increasing thereafter. The major driver for such an increase is the grey iron foundries (SNAP 030103), a sector that in 2019 contributed 20 % of the total emissions. Burning of agricultural waste (SNAP 090700) and exhaust emissions from agriculture machinery (SNAP 080600) contribute 17 % and 13 %, respectively. This compound commonly results from combustion processes, therefore, is expected to be detected in several combustion activities.

The total emissions of propene (or propylene) have not significantly changed between 2010 and 2019, although there is a constant increase in SNAP 01 emissions. The increase from this sector is mainly driven by the rapid and almost constant increase of emissions from SNAP 010103 (Public power - Combustion plants ≥20 and <50 MW) due to the increase in biomass consumption (MITERD, 2021a) and SNAP 010405 (Solid fuel transformation - Stationary engines). The biggest contributor source is Polypropylene production (SNAP 040509), representing around 51 % of the total emissions.

Toluene and xylenes show similar dominating sources and trends. The two species are dominated by the solvent sector, mainly paint applications on wood - SNAP 060107 (24 % and 37 %, respectively), other industrial paint applications - SNAP 060108 (24 % and 19 %, respectively); and road transport, mainly passenger cars. This results from the use of toluene and xylenes in paints and coatings due to their high solvency power and as blending components in road transport petrol fuels. The total emissions of both species present a significant drop between 2010 and 2012 (−22 % and −23 %, respectively) and thereafter up to 2019, an almost negligible reduction (−4 % and −3 %, respectively). The large drop between 2010 and 2012 is mainly linked to s decreases in emissions from industrial paint applications (SNAP 060108).

**Fig. 4:**
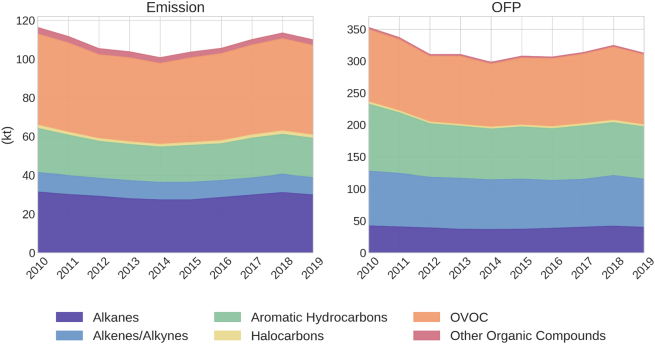
Evolution of emissions (kt) and OFP (kt) by the chemical group between 2010 and 2019 (June to August).

The speciated NMVOC emissions were classified according to six major chemical groups, namely, alkanes, alkenes/alkynes, aromatic hydrocarbons, halocarbons, and oxygenated volatile organic compounds (OVOC). Note that around 6 % of the total speciated emissions could not be grouped into any of the six categories since they were reported as unknown or similar. Fig. 4 shows the evolution of total emissions and OFP by the chemical group between 2010 and 2019.

OVOC represent the largest contribution to total emissions throughout the analysed period (42 % in 2019), followed by alkanes (30 % in 2019) and aromatic hydrocarbons (18 % in 2019). The contributions to OFP differ substantially, as the chemical groups with the highest reactivity values are alkenes/alkynes, aromatic hydrocarbons and OVOC. For example, alkenes/alkynes significantly increase their contribution, from 8 % in emissions to 23 % in OFP, and the low reactivity of alkanes reduces in more than half its contribution to OFP relative to emissions (30 % versus 13 %). Over the study period, the contributions to OFP of the different chemical groups have not significantly changed, the main contributors in 2019 being OVOC (36 %), aromatic hydrocarbons (26 %) and alkenes/alkynes (23 %).

### 3.2 Speciated emission inventory comparison

The speciated NMVOC emission inventory derived in this study was compared against the Copernicus Atmosphere Monitoring Service European Regional air pollutant emission inventory (CAMS-REG-APv5.1). CAMS-REG-APv5.1 provides gridded emission information at the European scale to be used in air quality production and forecasting systems. The CAMS-REG-APv5.1 inventory covers the years from 2000 to 2018 for multiple pollutants and sectors following the GNFR classification system, with a spatial resolution of 0.05 × 0.1 degrees (Kuenen et al., 2022). Besides reporting total annual gridded emissions, CAMS-REG-APv5.1 also provides chemical speciation profiles that vary per country, sector and year, and that allow splitting total NMVOC emissions into the 25 individual NMVOC groups as set by the Global Emissions InitiAtive (GEIA) (Huang et al., 2017).

We combined the total Spanish NMVOC emissions and associated speciation profiles provided by CAMS-REG-AP to compute sector-dependent speciated emissions and compare them against the results obtained in this work. To be able to compare both inventories, we mapped the species from our speciated inventory to the 25 GEIA NMVOC groups (i.e., v1 to v25). This mapping can be found in the Supplementary material in Table S3 and, in Table S4, the corresponding GEIA group name. Due to difficulties to map some species (e.g., species classified as unknown), 4.9 % of the total speciated emissions could not be mapped. Furthermore, to compare the sector contribution, we mapped the SNAP sectors to the GNFR categories following the mapping table provided by CEIP EMEP (2019). The GNFR sectors G (Shipping) and L (AgriOther) were not accounted for in the comparison as they are linked to the SNAP emission sectors that we excluded in this work (see Section 2.1). The comparison was made for 2018, as it is the last available year for CAMS-REG-APv5.1.

**Fig. 5:**
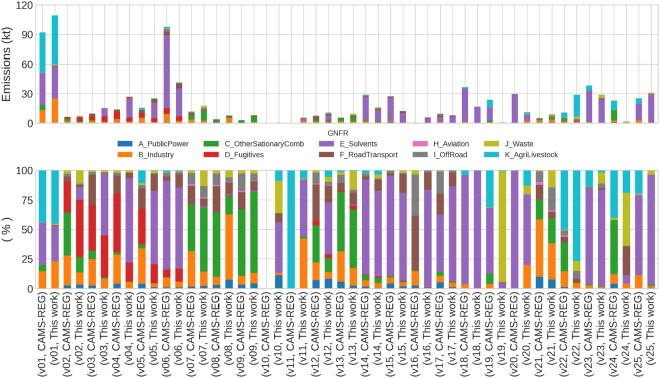
Comparison between the speciated inventory developed in this work and the CAMS-REG-APv5.1 inventory for the year of 2018 by GNFR sector and GEIA NMVOC group (v1 to v25) in terms of total emissions (kt/year) (top) and relative contribution (%) (bottom).

Fig. 5 shows the comparison of the speciated NMVOC emission inventory developed in this work and CAMS-REG-APv5.1. The total NMVOC emissions are 457 kt/year (this work) and 528 kt/year (CAMS-REG-APv5.1) (13 % differences). While both inventories use as a basis the Spanish officially reported emissions under the CLTRAP, this difference is mainly explained by the different versions of officially reported data considered in each case and recalculations that have occurred between them. While in this work, we consider official emission data as reported in 2022, CAMS-REG-APv5.1 builds on emission data as reported in 2020. Moreover, the discrepancy in the totals is also partially explained by the incomplete mapping from individual species to the 25 GEIA NMVOC groups mentioned earlier.

In both inventories, the GEIA NMVOC groups v01- Alkanols (alcohols) and v06 - Hexanes and higher alkanes are the ones reporting the largest emissions. For these two species, the sectoral contributions to the overall emissions are also consistent between inventories, with the agricultural livestock (GNFR K) and solvent (GNFR E) sectors being the main contributors to v01 and v06, respectively. Despite these agreements, we see that the total amount of v06 emissions reported by CAMS-REG-APv5.1 are approximately two times larger than emissions estimated in the present work (98 kt versus 41 kt). A good agreement both in terms of total emissions and sectoral contributions can also be observed for v23 – alkanones (ketones) and v25 – other NMVOC, the main contributor being, in both cases, the solvent sector (>75 %). Significant contributions from fugitive fossil fuel emissions (GNFR D) are reported in both datasets for v02 - ethane, v03 – propane and v04 - butane, CAMS-REG-AP tending to present slightly larger shares. Other discrepancies can be found, for instance, in the case of ethers (v19), for which our inventory reports only a total of 0.7 kt, the main source being waste treatment and incineration (GNFR J) (93.6 %), while CAMS-REG-APv5.1 reports much larger emissions (24 kt), mainly coming from the solvent (GNFR E) (55.7 %) and livestock (GNFR K) (31.5 %) sectors. For acids (v24), the emissions reported by CAMS-REG-APv5.1 are higher (23 kt) than for our inventory (1.7 kt). For CAMS-REG-APv5.1, the main sources are (GNFR C) (45.8 %) and livestock (GNFR K) (40.3 %), while according to this are waste management (GNFR J) (45.1 %), road transport (GNFR F) (24.3 %), and livestock (GNFR K) (19 %).

CAMS-REG-APv5.1 assumes that no isoprene (v10) is emitted from anthropogenic sources, while in our inventory, there is a small amount (0.5 kt) emitted by multiple sectors, mainly solvents (GNFR E) (42.4 %) and waste management (GNFR J) (27.5 %). When looking at the other less emitted species, the contribution of the main sectors have a bigger variability between both inventories. This is caused by assuming different assumptions and simplifications combined with different speciation profiles.

### 3.3 OFP sensitivity analysis

**Fig. 6:**
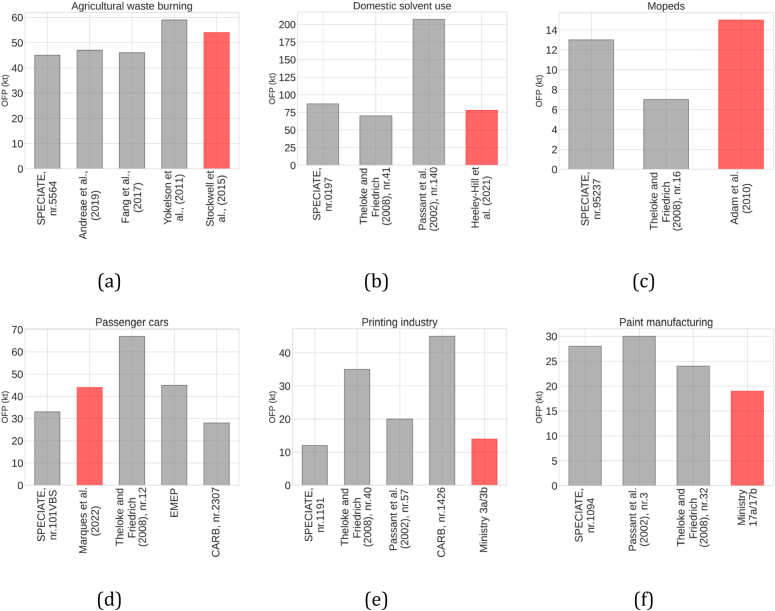
OFP (kt/year) estimated for selected SNAP activities using different speciation profiles in 2019. For each sector, the bar highlighted in red represents the profile used in this work.

As shown in Section 2.1, the selection and application of chemical profiles can lead to large uncertainties in estimating speciated NMVOC emissions. For this reason, we performed a sensitivity analysis to quantify the importance of the speciation profile selection on the estimation of OFP. For each SNAP activity described in Section 2.1, OFP values were calculated considering the different speciation profiles collected from the literature. Fig. 6 summarises the resulting OFP values obtained for each SNAP sector and speciation profile. For each sector, the profile used in this work is highlighted in red.

For agricultural waste burning (Fig. 6a), only slight variations are observed among OFP values obtained from each profile. The profiles from SPECIATE (45 kt), Andreae (2019) (47 kt) and Fang et al. (2017) (46 kt) report very similar OFP values. The profiles from Yokelson et al. (2011) and Stockwell et al. (2015) show slightly higher values (59 kt and 54 kt, respectively) mainly due to the large share of formaldehyde considered in their profiles, which has a high reactivity (7.16).

For domestic solvent use (Fig. 6b), a significant discrepancy is observed between the OFP derived from Passant (2002) (207 kt) and the other profiles (SPECIATE, Heeley-Hill et al. (2021) and Theloke and Friedrich (2007)), which report OFP values more than two times lower, between 87 and 70 kt. For the profiles from SPECIATE and Theloke and Friedrich (2007), the discrepancy is explained by the reactivity of the dominating species; the MIR value of isopropanol (0.64) is two times lower than that of butane (1.33), which is the primary specie in Passant (2002). For Heeley-Hill et al. (2021), the discrepancy is explained by the overall low reactivity of the major species, mainly from propane (0.56) and acetone (0.34), which contribute 28 % of the profile.

For mopeds (Fig. 6c), the profile from SPECIATE (13 kt) and Adam et al. (2010) (15 kt) show similar values, while the profile from Theloke and Friedrich (2007) shows an almost two times lower OFP value (7 kt) due to the low percentages of highly reactive species, e.g., ethylene and propylene, present in the profile.

For passenger cars (Fig. 6d), there are large discrepancies among resulting OFP values. The profile from Theloke and Friedrich (2007) shows the highest OFP (67 kt), mainly due to the high contribution from ethylene, followed by the profiles from Marques et al. (2022) (44 kt) and EMEP (33 kt). The profiles from SPECIATE (33 kt) and CARB (2022) (28 kt) show the lowest values, mainly because of the larger fraction of less reactive species (e.g., benzene, ethanol, isopentane).

The printing industry (Fig. 6e) shows very large discrepancies. The profile from CARB (2022) has the highest values (45 kt), followed by Theloke and Friedrich (2007) (35 kt). On the other hand, the OFP obtained from Passant (2002), the ministry VOC reports, and SPECIATE are 2 to 4 times lower. This can be explained by the high reactivity of the major species for Passant (2002), e.g., naphtha (3.13) and methyl isobutyl ketone (3.81), and by the reactivity of toluene (4.02), which is 39 % of the profile for Theloke and Friedrich (2007).

For paint manufacturing (Fig. 6f), similar OFP values are obtained when considering Passant (2002) (30 kt), SPECIATE (28 kt) and Theloke and Friedrich (2007) (24 kt), while the profile derived from the Spanish VOC reports a smaller OFP (19 kt).

Overall, it is observed that the impact of speciation profiles on OFP estimation is not homogeneous and can greatly vary among emission sources. For certain sources, such as agricultural waste burning or paint manufacturing, large differences between speciation profiles lead to relatively low differences in the resulting OFP because the dominant species of each profile have similar MIR values, which translates into a reduced impact on the estimated OFP. On the other hand, for other activities, such as printing, differences between profiles imply large differences between OFP values.

### 3.4 Sectoral contribution to ozone formation potential

**Fig. 7:**
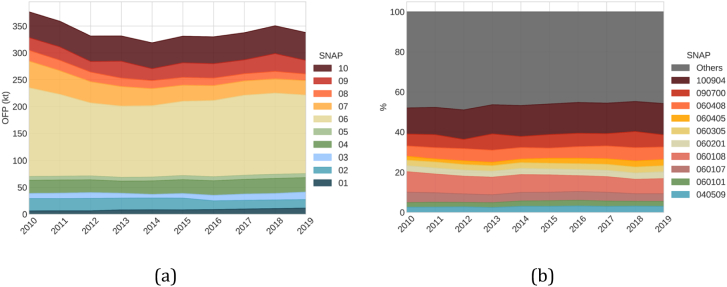
Evolution of the sector contribution (between June and August) for 2010–2019, where: a) contribution per SNAP group to total OFP (kt), and b) contribution (%) of the top ten SNAP activities.

Fig. 7a shows the evolution of the total OFP estimated for June to August and the contribution per SNAP group between 2010 and 2019. During this period, NMVOC emissions (−1.4 %) decreased less than total OFP (−10 %), indicating that the reductions occurred for sectors emitting more reactive species. Despite that, the reductions of NMVOC are quite low when compared to other pollutants, e.g., NOx decreased by −29 % over the same period.

When analysing the evolution trend of total OFP since 2010, a considerable reduction is observed until 2014 (−15 %). The main driver in the total reduction is SNAP 06 - Solvent and other product use (−9 % of the total) and SNAP 07 - road transport (−3 %). These reductions can be explained by the economic crisis that began in 2008 and finished in 2014 (Castellanos and Boersma, 2012; Sobrino and Monzon, 2014), which heavily impacted the manufacturing industry sector as well as the livelihood and level of consumption of Spanish citizens. Moreover, the road transport sector has been continuously reducing its emission due to the introduction of new EURO vehicle standards and the strong dieselisation effect on the vehicle fleet composition, which overall implies a reduction of the average NMVOC emission factor (EF) for this sector, as the NMVOC EF for diesel vehicles are approximately one order of magnitude lower than the ones reported for gasoline-powered cars (EMEP/EEA, 2019a). From 2014 on, the OFP rebounded for most sectors (+11 % in total from 2014 to 2018), only with a decrease between 2018 and 2019 (−3.7 %).

The SNAP groups show different OFP evolution trends over the years. SNAP 01 - Combustion in energy and transformation industries has constantly increased between 2010 and 2019, driven by a factor of 3 enhancement in biomass consumption for public electricity generation, increasing the OFP by 90 %. Meanwhile, SNAP 02 - Non-industrial combustion plants have diminished, mainly due to emission reductions from commercial and institutional stationary gas turbines - SNAP 020104 (−62 %) and turbines - SNAP 020105 (−54 %). The total OFP from this sector in 2019 was 30 % lower than in 2010. SNAP 03 - Combustion in the manufacturing industry reached a minimum in 2014 and increased thereafter. In 2019 the sector emitted 113 % more than in 2014 and 55 % more than in 2010 due to the increase from the paper-mill industry (118 % more in 2019 than in 2010). SNAP 04 - Production processes reached a minimum in 2013, increasing thereafter. In 2019 the OFP for this sector was +10 % higher than in 2010. SNAP 05 - Extraction and distribution of fossil fuels oscillate yearly, but in 2019 the levels were 10 % higher than in 2010. SNAP 06 reached a minimum in 2014 and increased thereafter, with a slight decrease in 2019. Overall, SNAP 07 has diminished constantly (a reduction of −36 % by 2014) due to restrictions on passenger cars, despite the slight increase (+2 %) in 2019 relative to 2018. SNAP 08 - Other mobile sources and machinery have constantly decreased, reaching −38 % in 2019 relative to 2010. SNAP 09 - Waste collection, treatment and disposal activities show a strong variability over the years, and SNAP 10 - Agriculture and farming show a constant increase since 2013 (+11 % in 2019).

Fig. 7b shows the trend of the top ten activities contributing to OFP. These activities account for 54 % of the total OFP in 2019, of which 30 % comes from SNAP 06. Major activities are SNAP 100904- Manure management (16 %), followed by SNAP 060108 - industrial paint application (8 %), and SNAP 090700 - agricultural waste burning (6 %). Manure management kept growing, and in 2019 it was 8 % higher than in 2010. Industrial paint application (excluding application on cars, construction, domestic use, coil, boats and wood) has been decreasing (−34 % between 2010 and 2019). Agricultural waste burning is an activity that varies greatly each year. SNAP 060408 - domestic solvent use is a sector that should not be overlooked due to its importance to the total OFP, and in 2019 was +5 % higher than in 2010.

### 3.5 Spatial variability

**Fig. 8:**
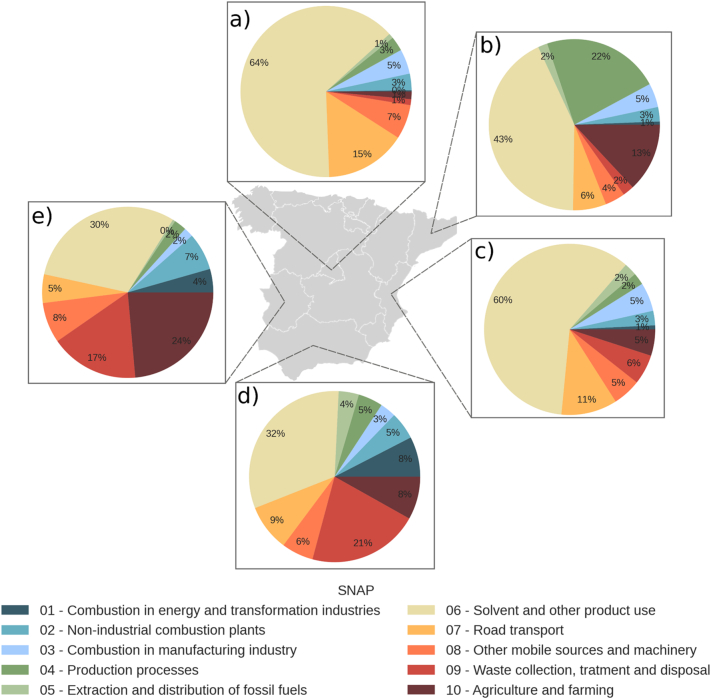
Sector contribution for autonomous communities (NUTS 2) between June–August for 2019, where: a) Madrid, b) Catalonia, c) Valencia, d) Andalusia, and e) Extremadura.

The Spanish official emission inventory is reported at the national level, but for informational purposes, approximated disaggregation is provided by autonomous communities (NUTS 2) for greenhouse gas (GHG) emissions. Disaggregation for air pollutants has been provided by MITECO for this work. Fig. 8 shows the contribution of each SNAP sector to OFP for 2019 for five different autonomous communities: Catalonia, Madrid, Valencia, Andalusia and Extremadura. These regions of Spain have been identified as the ones recording the most frequent and intense O_3_ pollution episodes (Massagué et al., 2022; Querol et al., 2016).

Madrid and Valencia show similar sector contributions, the dominant source being the solvent sector (SNAP 06) with 64 % and 60 %, respectively. In Catalonia, the solvent sector is also the dominating source (43 %), followed by industrial processes (SNAP 04) (22 %), where the chemical industry has the major contribution. In Andalusia and Extremadura, which can be defined as agricultural regions when compared to the previous ones, the role of the solvent sector is lower (30–32 %). In these regions, the focus turns to waste management (SNAP 09) (17–21 %) which is dominated by agricultural waste-burning activities, and agriculture (SNAP 10) (24 % in Extremadura), which is dominated by manure management activities.

The different autonomous communities show common dominating sources, as identified previously in Section 3.4, such as: (i) domestic solvent use (060408), which appears within the top 5 contributors in all the autonomous communities, with contributions ranging between 4.6 % and 10.4 % of the total OFP; (ii) other industrial paint applications (060108), which has a significant contribution in Madrid (14.8 %), Valencia (11.2 %) and Catalonia (9.2 %); iii) burning of agricultural waste (090700), which greatly contributes in less urbanised regions, i.e. Andalusia (20.4 %) and Extremadura (16.3 %), and to a lesser extent, in Valencia (5 %) and (iv) manure management operations (100904), which has a significant impact in Extremadura (23.6 %), Catalonia (13 %), and Andalusia (7.7 %).

Each autonomous community also presents specific problems. Results in Madrid indicate a large contribution from the transport sector (i.e., passenger cars(10 %)) and from the solvent use in the industry sector, including metal degreasing (6 %) and the use of glues and adhesives (5 %). In Catalonia, industrial production processes contribute to 22 % of the total OFP, the production of polypropylene (SNAP 040509) being the main driver (13 %). Paint manufacturing (SNAP 060307) also has a significant contribution (3.5 %). In Valencia, industrial paint applications show a significant contribution, mainly to wood (7.3 %) and the manufacture of automobiles (5.3 %). Several power plants (SNAP 010103) are in Andalusia, which contribute 6.2 % to total OFP in the region. Andalusia and Extremadura have a lot of activity regarding fat, edible and non-edible oil extraction (SNAP 060404), which comes mainly from olive oil. However, this activity has a bigger OFP relative contribution in Extremadura (9.4 %) than in Andalusia (2 %) due to the importance of other sectors in the latter.

### 3.6 Projections for 2030

In this section, we quantify the expected impact of the NMVOC emission abatement measures reported under the National Air Pollution Control Programme (PNCCA) (MITECO, 2019b) and the National Integrated Energy and Climate Plan (PNIEC) (MITECO, 2020) on future levels of OFP. We used the official emission projections reported by MITERD for 2030 under the CLRTAP convention in March 2021, which consider implementing the actions reported in the PNCCA and PNIEC. Based on this information and the methodology described in Section 2, the OFP for 2030 was estimated and compared to 2019 values for selected sectors representing 52 % of the total OFP, including the public electricity sector (SNAP 0101), coating applications (SNAP 0601), other solvent uses (SNAP 0604), the road transport sector (passenger cars, SNAP 0701; light duty vehicles, SNAP 0702; heavy duty vehicles and buses, SNAP 0703; mopeds and motorcycles <50 cm^3^, SNAP 0704; mopeds and motorcycles, SNAP 0705) and livestock manure management (SNAP 1009). Other sectors could not be quantified in this analysis due to the lack of detail of the sector classification provided in the CLRTAP emission projections template, which limits the direct mapping of sectors to specific speciation profiles and the corresponding MIR values.

**Fig. 9:**
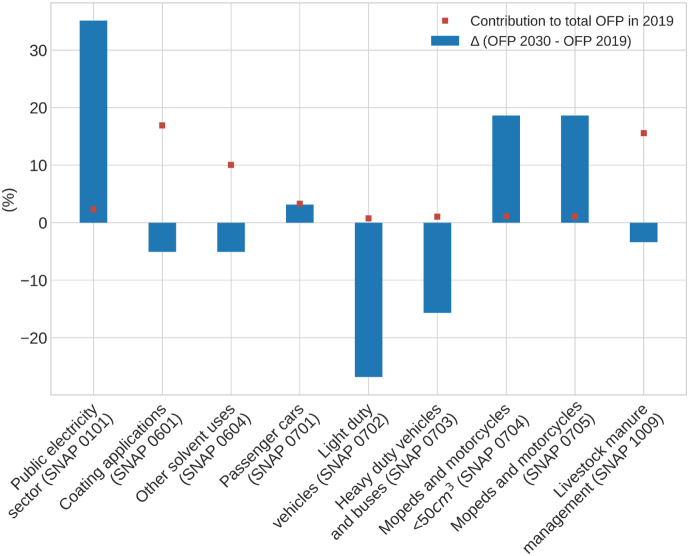
Variation of OFP (%) in 2030 compared to 2019 for selected sectors.

Fig. 9 shows the relative changes (%) in OFP between 2019 and 2030 for some SNAP activities. The contribution of each SNAP to total OFP in 2019 is also plotted for reference. According to the results, the total OFP is expected to increase slightly by 1.6 kt (+0.4 %) in 2030 when compared to 2019, while NMVOC emissions are expected to decrease by −11 % in 2030 with respect to 2019.

For the public electricity sector (SNAP 0101), an OFP increase from 30 kt in 2019 to 41 kt in 2030 (+37 %) is expected. This increase occurs despite the planned closure of all Spanish coal-fired power plants by 2030, and it is mainly driven by the expected increase in electricity production by biomass power plants (+77 %, corresponding to an increase of 14.7 kt), which will entail an increase of NMVOC emissions from this sector of +37 %. This significant increase will enhance the contribution of the sector to total OFP by 2030.

For activities related to the use of solvents (SNAP 0601 and SNAP 0604), and despite their large contribution to total OFP in 2019 (27 %), a limited impact is expected on 2030 OFP levels, with only a reduction of −4.5 kt. These results are in line with the almost negligible NMVOC emissions reductions expected for these activities (−5 %).

For the road transport sector (SNAP 07), variations of OFP between 2019 and 2030 are expected to be heterogeneous among vehicle categories. For passenger cars (SNAP 0701), an OFP increase of +3 % (1.4 kt) is expected, mainly due to the increase in gasoline vehicles and the decrease in diesel vehicles. For both light (SNAP 0702) and heavy (SNAP 0703) duty vehicles, strong decreases are expected (−27 % and −16 %), which will lead to reductions of OFP by 2030 of −2.7 kt and −2.2 kt, respectively. Nevertheless, these changes won't have a significant impact upon the overall OFP since their contribution is almost negligible (1.7 %). Opposite impacts are expected in mopeds (SNAP 0704) and motorcycles (SNAP 0705), with increases in the OFP of 2.8 kt (19 %) and 2.6 kt (19 %), respectively. Similarly to what is observed in the public electricity sector, the role of these two sources in terms of OFP contribution may become more relevant in the future when compared to the current status.

For the livestock manure management sector (SNAP 1009), a small reduction of −7 kt (−3.4 %) is expected by 2030 relative to 2019. Despite the projected reductions, this sector is still expected to be one of the major activities contributing to total OFP.

## 4 Conclusions

In this work, we developed a speciated and OFP-based NMVOC emission inventory for Spain from 2010 to 2019 to identify the main species and activities contributing to OFP. Emissions from >900 individual NMVOC chemical species were estimated based on Spanish official NMVOC reported emissions and a profile-assignment approach. State-of-the-art speciation profiles were compiled and compared, and the most appropriate were selected for a total of 153 anthropogenic emission sources. The resulting speciated emission inventory was compared against the CAMS-REG-AP speciated NMVOC emissions for 2018, showing an overall good agreement for the major species. The impact of individual NMVOC species and emission sources to OFP was estimated using the MIR approach. The analysis of this reactivity-based approach was performed both at the national level and also for the main Spanish regions with O_3_ problems. Additionally, official emission projections for 2030 were considered to quantify how planned abatement measures could impact OFP values and contributions for selected sectors in the near future.

Overall, the main activities contributing to O_3_ problems in Spain in 2019 are: (i) the solvent sector, mainly for multiple paint application (18 %), and the domestic solvent use (6 %), (ii) manure management (16 %), and (iii) agricultural waste burning (4 %). The impact of these sectors changes by autonomous communities. For example, in more urbanised areas, the solvent sector has a bigger impact, and in rural areas, manure management and agricultural waste burning gain importance.

The top 10 key reactive species contributing most to OFP, which account for 54 % of the total OFP, are ethanol, ethene, xylenes, propene, toluene, formaldehyde, 1,3-butadiene, styrene, n-butane, and cyclopentane, are within the major three chemical groups contributing to OFP in 2019, i.e., OVOC (36 %), aromatic hydrocarbons (26 %) and alkenes/alkynes (23 %). Our study, in line with others (Derwent et al., 2007; Fu et al., 2020; Liang et al., 2017; Ou et al., 2015), showed that targeting species using the reactivity-based control should be more efficient than the emission-based approach since a smaller effort is required to achieve similar OFP reductions. For example, to achieve a reduction of 80 % in OFP, tackling highly reactive species is more effective since it requires a smaller reduction of emissions (65 %) and a smaller number of species (32 species). In contrast, reducing emissions by tackling the highly emitted species will require controlling more species (44) and a larger reduction of emissions (84.5 %).

In 2030, the total OFP is expected to increase slightly by 1.6 kt (+0.4 %) relative to 2019, indicating that NMVOC variations occur among different sectors and species with different reactivity. The increase in total OFP is mainly due to the expected growth of biomass use for the public electricity sector (+77 % relative to 2019) but also to the road transport sector with a growth of passenger cars (+3 %), mopeds (+19 %) and motorcycles (+19 %).

### 4.1 Policy recommendations

We propose a set of recommendations based on the results obtained. Neither the implementation costs, i.e., political or economical, nor the feasibility of implementation are taken into account in this work.

• The use of paints and coatings is present in multiple activities and accounts for approximately 20 % of the total OFP in Spain. Toluene and xylene, which are among the top ten species contributing to OFP, are often used in these paints and coatings due to their high solvency power. The use of low-solvent or powder coatings could reduce overall NMVOC emissions by 40–65 % (EMEP/EEA, 2019c). Derwent et al. (2007) and Bockemeier (2020) identified that the substitution of toluene and xylenes in the solvent usage by high-purity paraffin such as isooctane n-heptane, n-octane could lead to significant O_3_ benefits.
• Agricultural waste burning, as previously mentioned, contributes significantly to total OFP in Spain (6 %) and gains even more relevance when looking at predominantly rural regions, e.g., in Andalusia (20 %) or Extremadura (16 %). In Spain, since April 2022, with the implementation of law 7/2022 of 8 of April, related to waste and contaminated soils for a circular economy (MPR, 2022), this activity became totally prohibited, being only legal under exceptional reasons when no other waste treatment is possible. The implementation of these laws is expected to reduce the emissions from this activity significantly. Therefore, the only recommendation is to monitor illegal activity.
• The domestic use of solvents is one of the main activities contributing to OFP. This sector includes the use of multiple products (i.e., cosmetic, household, construction/DIY, and car care products), which can be aerosol or non-aerosol products. Reduction strategies should primarily focus on aerosol products, as they are the major source of indoor air pollution. When reducing emissions from this sector, the strategy should not expect significant consumer changes but should focus on product composition. Sprays are the major sources of indoor air pollution, with about 80 % of all aerosol dispensers employing LPG as the propellant. Replacing this LPG propellant with compressed gas propellant, such as nitrogen, could lower annual NMVOC emissions by −30 to −52 % (Nourian et al., 2021).
• Manure management operations have a significant contribution to total OFP in Spain (15.7 %), especially in Extremadura (24 %), Catalonia (13 %), and Andalusia (8 %). As identified by Pedersen et al. (2021), more research is needed to accurately estimate NMVOC emission factors from this sector and, therefore, identify effective mitigation measures. However, some measures that are already defined in (EMEP/EEA, 2019e) can be the immediate covering of silage stores and minimisation of the area of silage available to feeding animals; and the use of high-quality feed with high digestibility, which reduces the amount of substrate for NMVOC formation.
• Apart from the aforementioned sectors, we identified that other activities could also play a significant role in specific regions. For example, in Catalonia, the chemical industry (21 %), mainly the production of polypropylene (13 %), greatly impacts OFP; in Andalusia, the public power production (7 %), although a reduction is expected due to the planned shutdown of coal plants by 2030; and, in Extremadura, the fat and oil extraction (9 %), which is a significant source of hexanes. For reductions in these sectors, the application of technologies to control fugitive emissions and available modern abatement methods are advised. For instance, to reduce hexane emissions associated with the processing of edible oil using solvent extraction methods, Kumar et al. (2017) shows that coupling green solvents with green technologies such as aqueous-assisted enzyme extraction could ensure oil quality and protein extraction but also reduce the environmental impact.

Regarding future projections, the expected increase in NMVOC emissions from biomass combustion in power plants by 2030 cannot be neglected and should be carefully addressed as it is expected to largely impact OFP. Therefore, applying VOC emission controls to these future plants is strongly advised. On the other hand, the transport sector shows a growing tendency in emissions from petrol-fuelled mopeds and motorcycles, which mainly operate in urban areas. The promotion and consolidation of electric mobility models such as the motosharing can help reduce emissions from this sector.

### 4.2 Limitations & uncertainties

The speciated and OFP-based emission inventory developed in this work is mainly based on three elements, including: i) officially reported NMVOC emissions, ii) chemical speciation profiles, and iii) MIR values associated to each individual species. The uncertainties associated with each one of these data sets lead to uncertainties in the results presented in this work.

Concerning official reported inventories and according to the Spanish Informative Inventory Report (MITERD, 2021a), NMVOC emission estimates in Spain are thought to have an uncertainty of about ±51.9 %. This uncertainty is much larger than the one estimated for other species, such as NOx (±16.2 %) or SOx (±18.6 %). This is mainly related to the large uncertainty in NMVOC emission factors (EF) associated to certain key sectors such as manure management (±300 %), coating applications (±58 %) and domestic use of solvents (±67 %). Moreover, the uncertainty introduced into the trend in total national emissions is around 15.9 %, with sectors having a more relevant uncertainty, such as the food and beverages industry (15.2 %), chemical products (11.0 %), and coating applications (9.2 %).

Several limitations arise when using chemical speciation profiles to disaggregate total NMVOC emissions into individual species. The sensitivity study performed in this work showed that speciation profiles reported by different databases for the same pollutant activity could greatly vary due to multiple factors, including year and location, types of fuel, technologies and processes included, among others. The selection of the chemical speciation profile can also play a crucial role in the estimation of the OFP for a specific activity. The estimated OFP for a specific source can differ up to 4 times depending on the speciation profile considered. In the present work, profiles derived from Spanish databases and European works were prioritised. Nevertheless, for 93 out of the 153 SNAP activities, profiles had to be derived from the USA SPECIATE database due to the lack of information. This gap indicates the need to develop a European NMVOC speciation profile database, following the efforts done by Pernigotti et al. (2016) to develop a European particulate matter source profile database. Furthermore, other uncertainties when working on speciated NMVOC emissions originate from the lack of detailed information, e.g., scarce long-term measurements of NMVOC compounds, limited efforts on updating emission factors, and where the national reporting only includes total NMVOC emissions and no information on speciation (Huang et al., 2017; von Schneidemesser et al., 2016).

Lastly, although the OFP method has been widely used, there are, however, some significant limitations such as (Council et al., 1999): the method does not account for potentially different yields of peroxy radicals formed from different species, the different reactive pathways these peroxy radicals can take once they are produced, and the varying tendency of VOC to enhance or inhibit radical levels, and thus influence the contribution of other VOC species to O_3_ formation. The MIR is quantified under specific conditions which may not be representative of ambient conditions. Furthermore, the value is calculated without considering multiple-day effects and is not calculated for all the compounds. Another limitation of this method is that it only quantifies the OFP from primary emissions but excludes the impact of secondary compounds formed in the atmosphere. This becomes especially relevant for OVOC species such as formaldehyde, which is a product of the ozonolysis of many halogenated VOC, and several works have identified its secondary production as the predominant source (i.e., 92 ± 4 % of total) (Parrish et al., 2011; Su et al., 2019). Consequently, the impact of this and other OVOC species on OFP may be underestimated in the present study.

The activities identified in this study as main contributors to OFP were derived from an analysis done at the national and NUTS-2 level. Performing an analysis at a higher spatial resolution (e.g., municipality or city-level) could reveal some other sources of importance. This includes, for instance, activities from the petrochemical industry in Huelva (Andalusia), which have been identified as a significant contributor to O_3_ acute episodes close to the city of Sevilla (Massagué et al., 2021), or the role of fugitive NMVOC released during the refuelling of cars in petrol stations, which have also been pointed out as key contributors to urban O_3_ levels in previous studies (Huy and Kim Oanh, 2020).

In general, O_3_ formation is most sensitive to NMVOC emissions for VOC-limited regimes, which are most commonly found in urban areas. So, while tackling NMVOC sources based on reactive-based strategies can be more efficient, depending on the regime, it can have a limited impact. Therefore, the recommendations provided in this study should be combined with other measures tackling other O_3_ emission precursors, mainly NOx. As observed during the summer of 2020 and 2021, the Mediterranean region of Spain met the EU O_3_ target values for the first time in all the recorded periods (Querol et al., 2021; Targa et al., 2022). Reductions occurred in emissions from road traffic due to COVID-19 mobility restrictions may not fully justify the marked decrease as, during the summer of 2020 and 2021, the decreases were only −20 % and − 10 % when compared to business-as-usual levels (Guevara et al., 2022). However, during these periods, emissions from cruises and air traffic activity were drastically lowered. This proves the need to tackle both NMVOC and NOx precursors when designing emission control strategies in order to effectively reduce O_3_.

### 4.3 Future works

Future works include the evaluation of the computed NMVOC speciated emissions by comparing modelled NMVOC concentrations against available observations. To achieve this objective, we plan to use the results of the present work to update the speciation profile database currently considered in the HERMESv3 emission model (Guevara et al., 2020), a bottom-up emission model that estimates anthropogenic emissions at high spatial (up to 1 km × 1 km) and temporal (hourly) resolution for air quality modeling. The resulting gridded emissions will be used to run air quality simulations over Spain with the MONARCH system (Badia et al., 2017) and finally compare the resulting NMVOC concentrations against measured levels collected from multiple Spanish observational campaigns (de Blas et al., 2019; Gómez et al., 2020; Yáñez-Serrano et al., 2021). Furthermore, the gridded speciation emission data derived with HERMESv3 will allow us to perform a more detailed spatial analysis of the speciated NMVOC emissions, i.e., at the province or city level, and identify or attribute greater importance to possible sources that could not be identified at the NUTS-2 level.

Finally, the speciated NMVOC inventory developed in this work can be used to assess the secondary organic aerosol potential (SOAP) from individual species and emission activities (McDonald et al., 2018; Srivastava et al., 2022) to assist the design of strategies to control particulate matter (PM) levels in Spain.

## CRediT authorship contribution statement

**K. Oliveira:** Writing – original draft, Conceptualization. **M. Guevara:** Writing – original draft, Conceptualization, Supervision. **O. Jorba:** Writing – review & editing. **X. Querol:** Writing – review & editing. **C. Pérez García-Pando:** Writing – review & editing, Supervision.

## Declaration of competing interest

The authors declare that they have no known competing financial interests or personal relationships that could have appeared to influence the work reported in this paper.

## Data Availability

Data will be made available on request.

## Appendix A Supplementary data

Supplementary tables S1–S4 Image 1

Supplementary figures S1–S2 Image 2
