# Supplementary material for: A new NMVOC speciated inventory for a reactivity-based approach to support ozone control strategies in Spain
Source: Sci Total Environ. 2023 Apr 1;867:161449. doi: 10.1016/j.scitotenv.2023.161449 (PMC9938404; doi:10.1016/j.scitotenv.2023.161449)
Supplement: Supplementary file 2 — Supplementary figures S1–S2 [file mmc2.docx]

## **Supplementary Material**

| 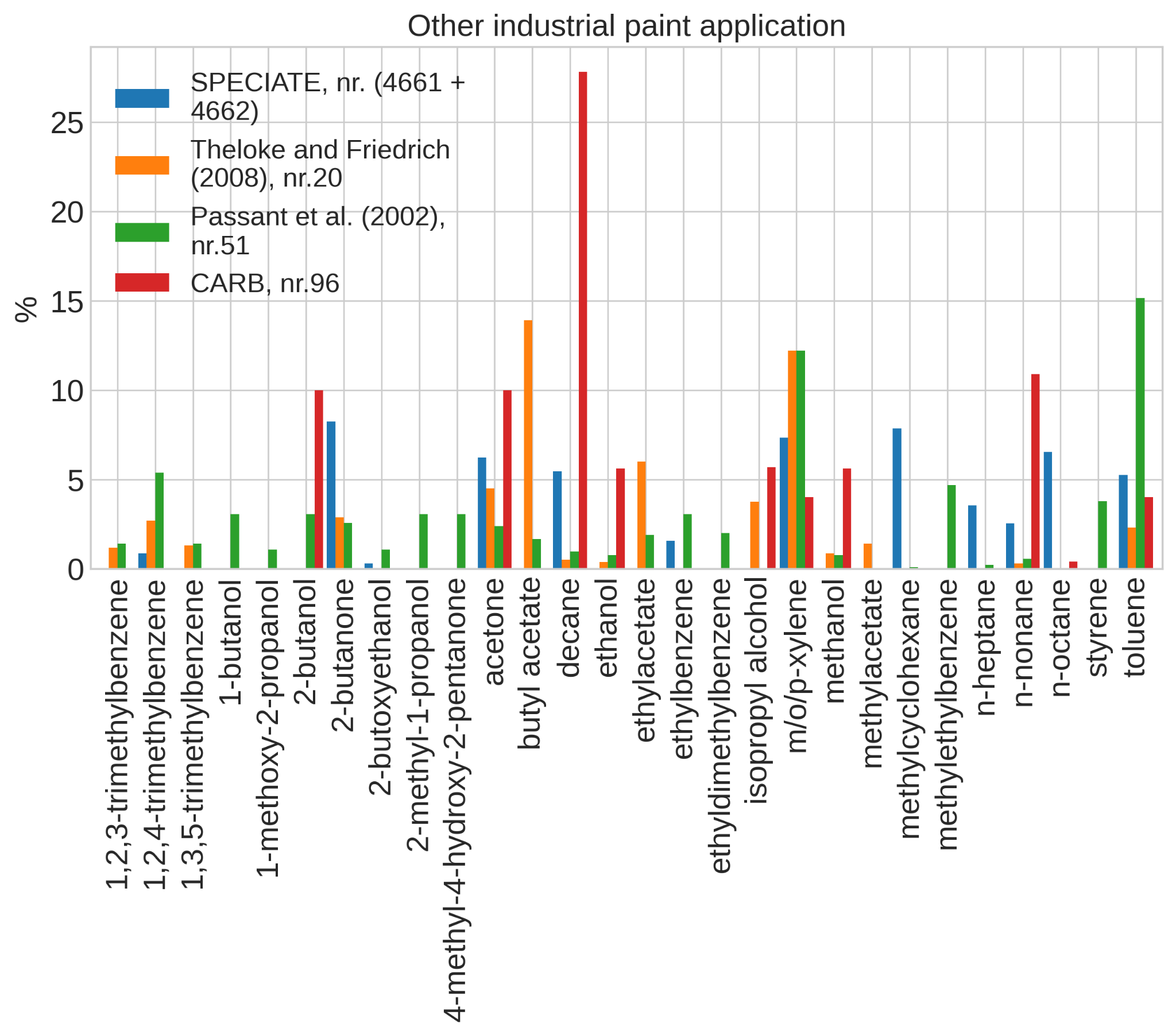 | 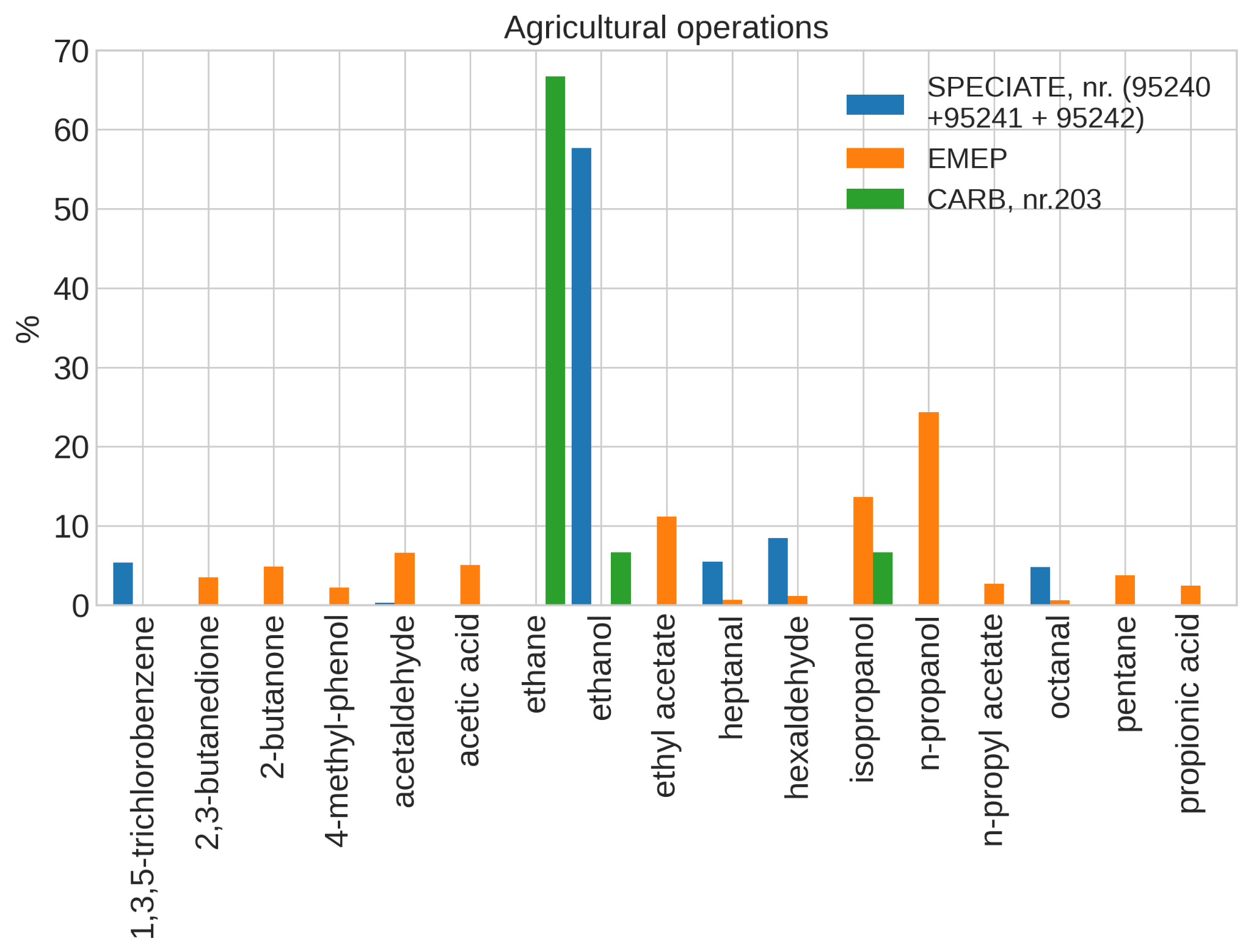 |
| --- | --- |
| 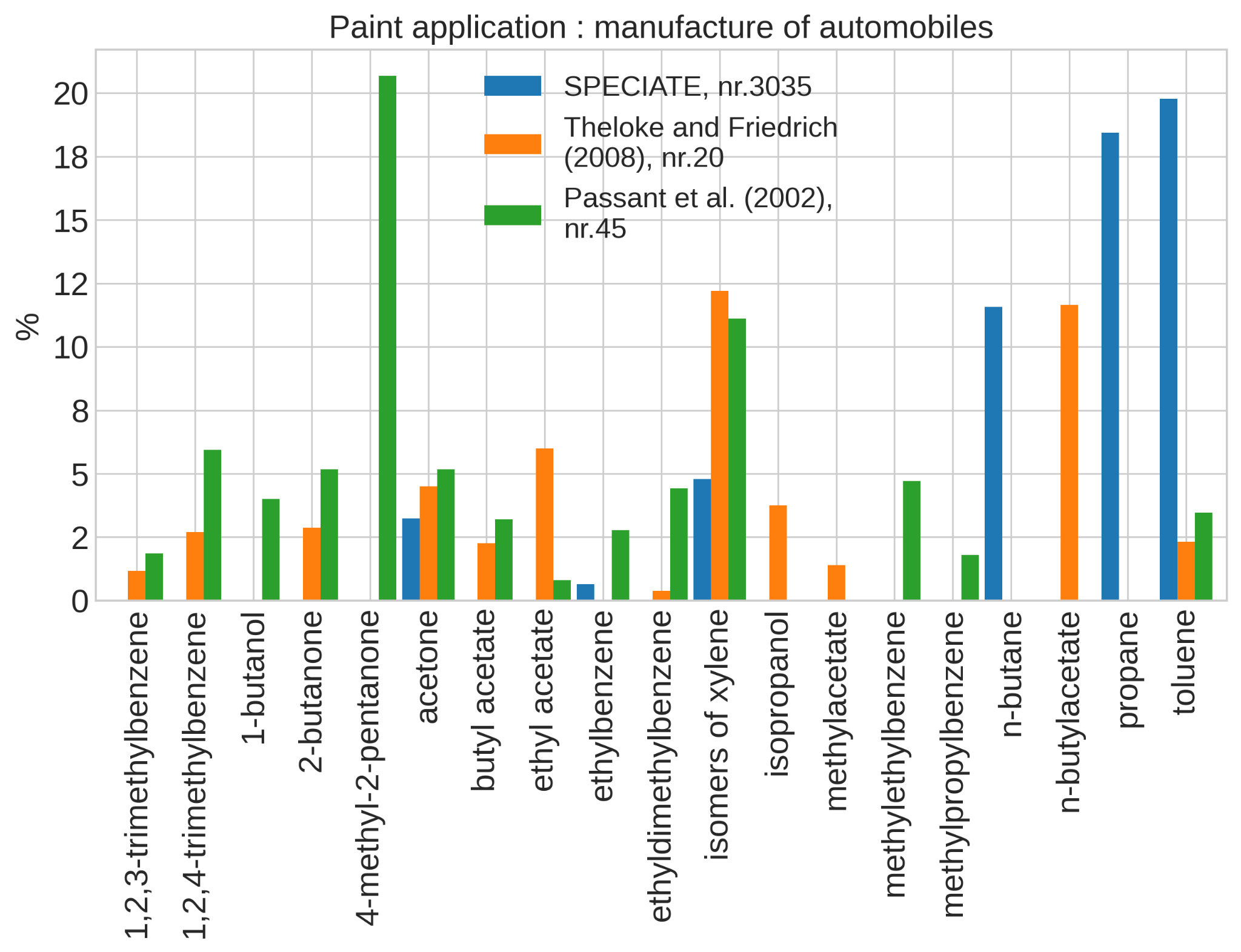 | 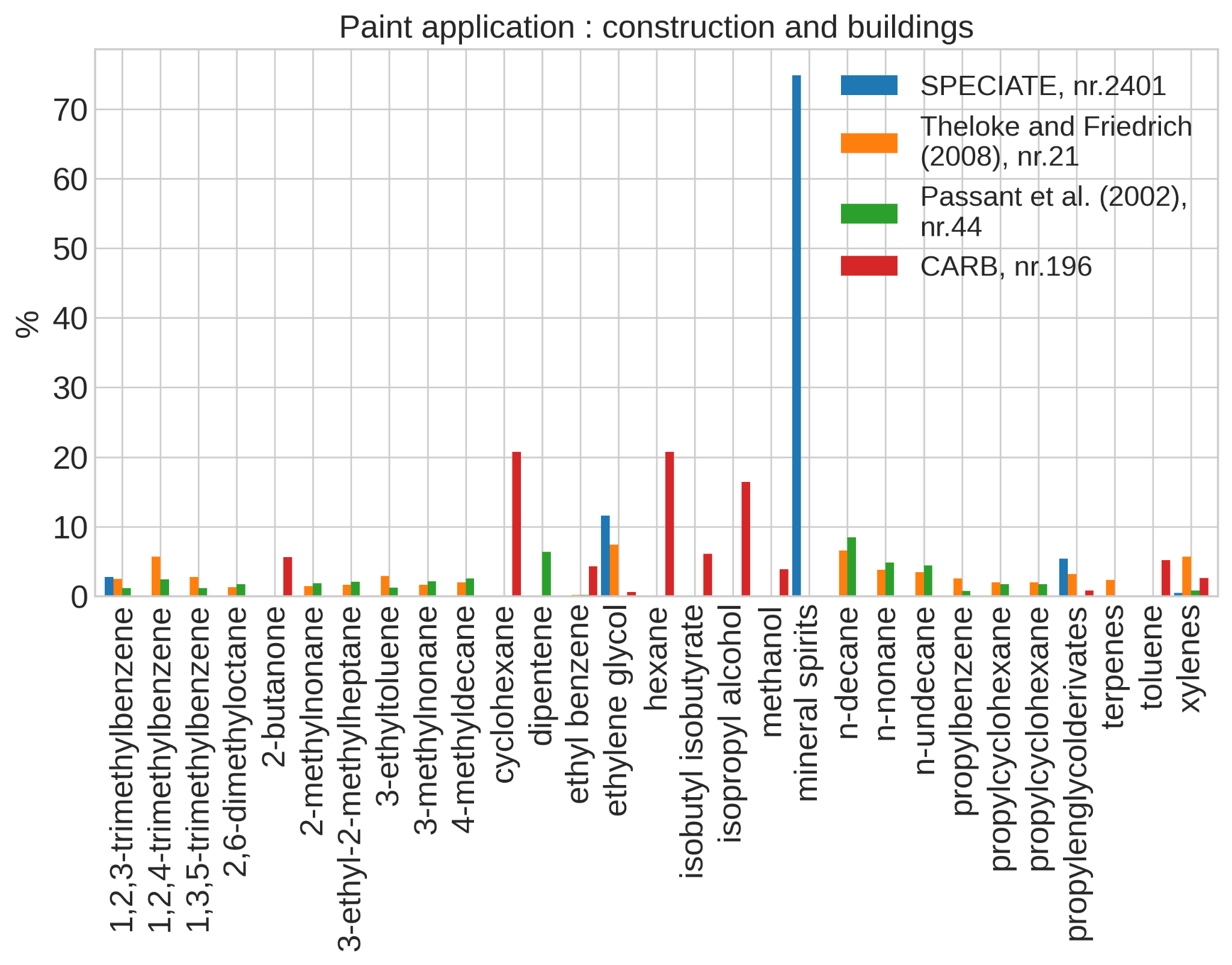 |

| 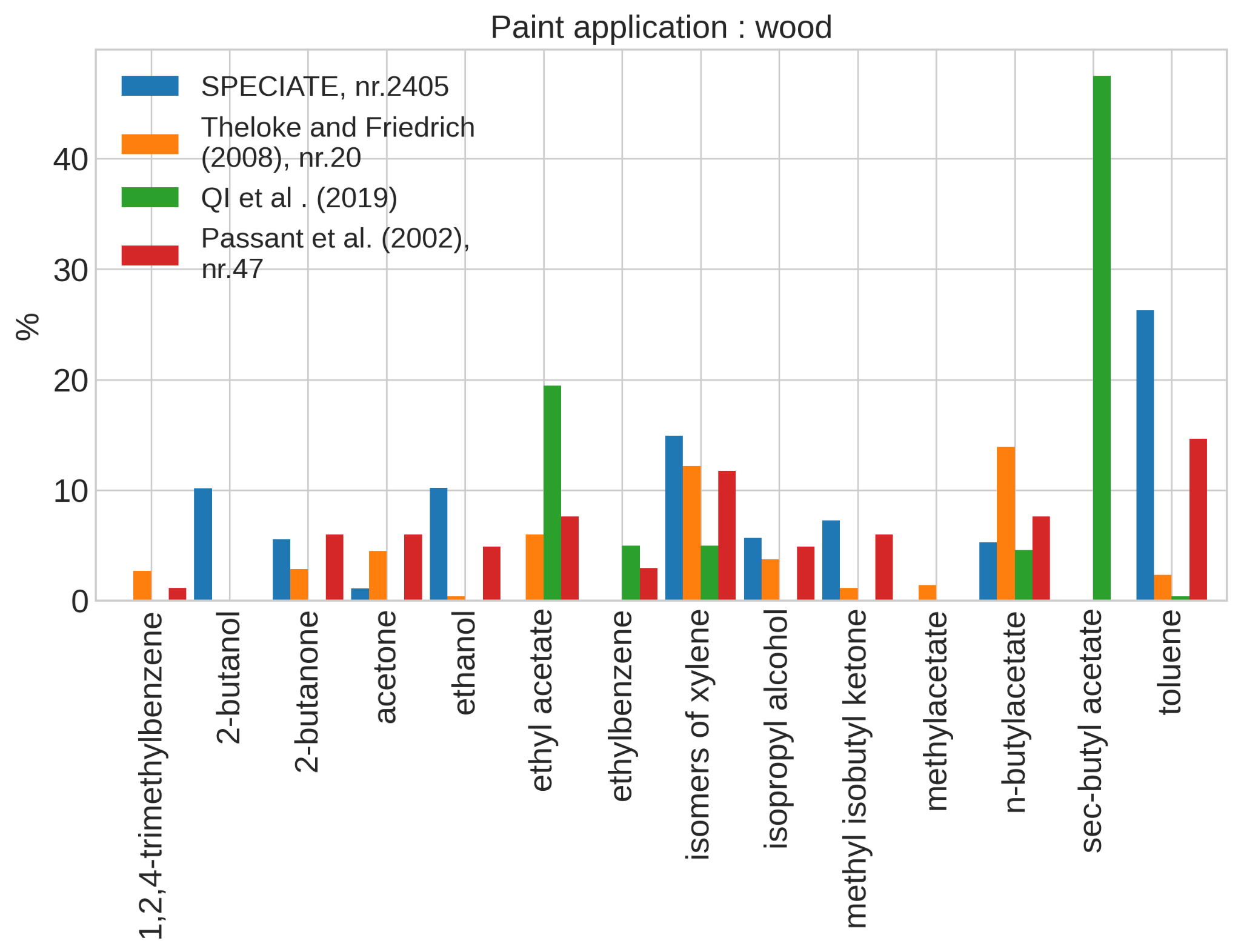 | 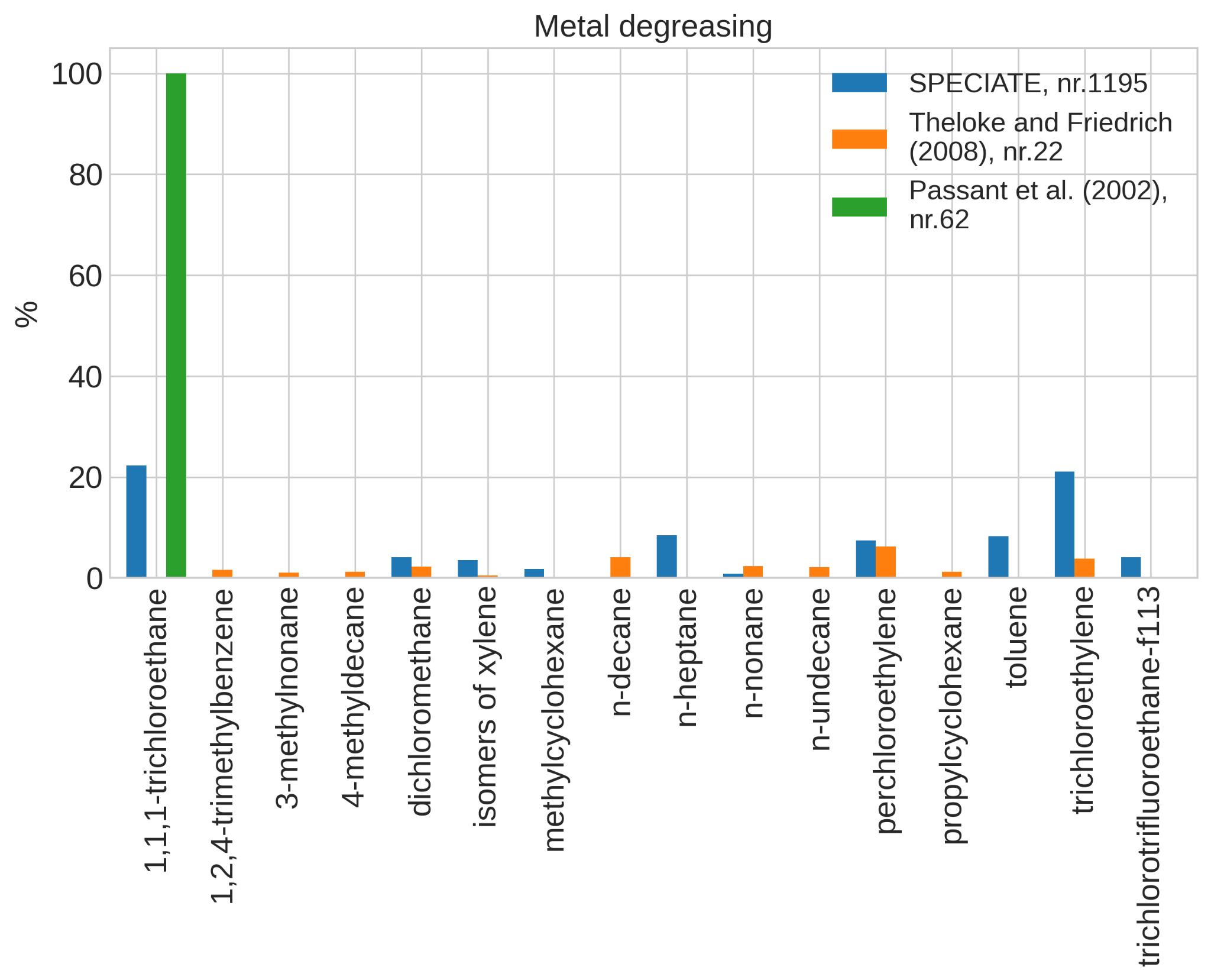 |
| --- | --- |
| 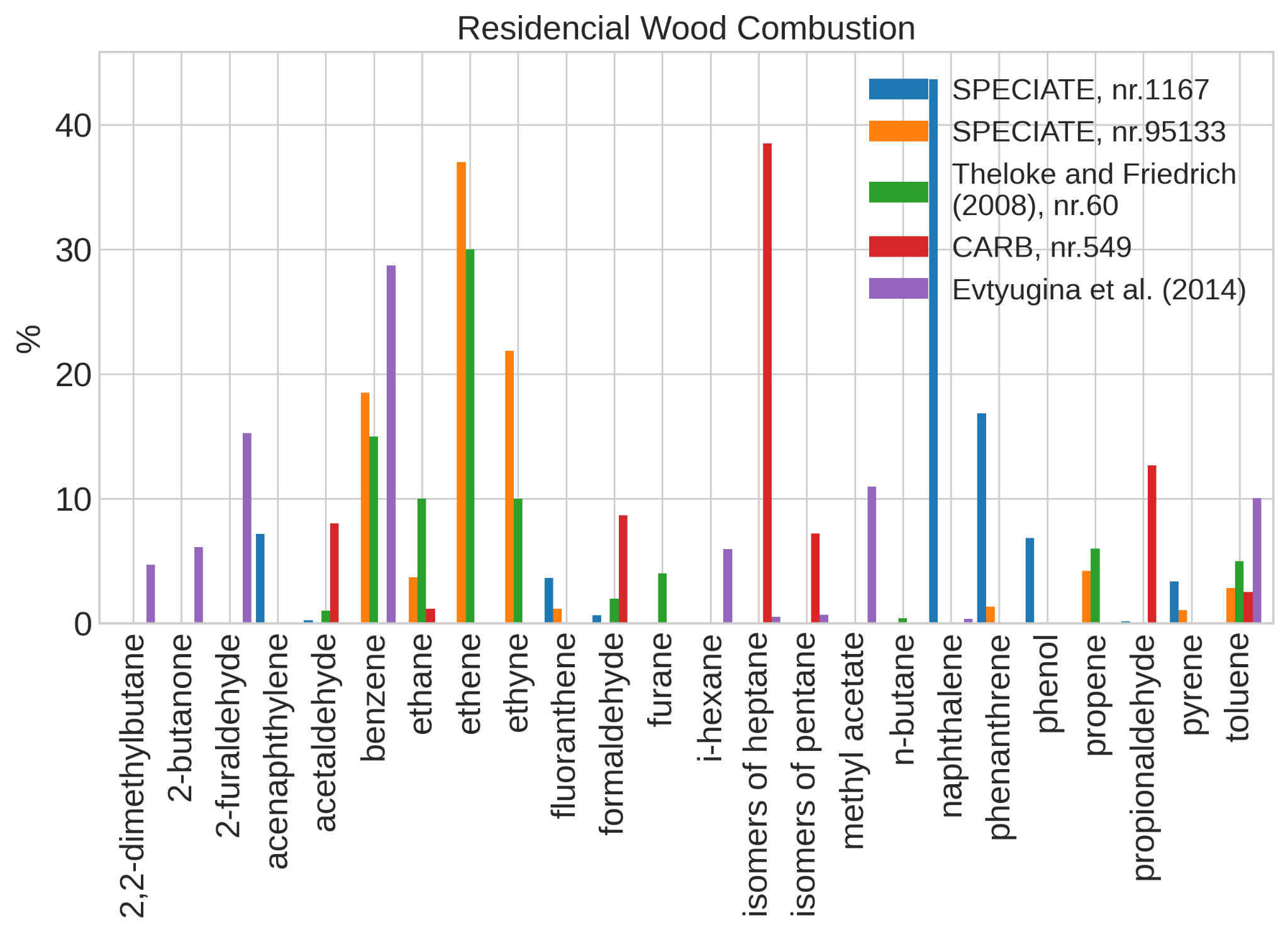 | |

Figure S1: Speciation profile comparison for specific activities.

| 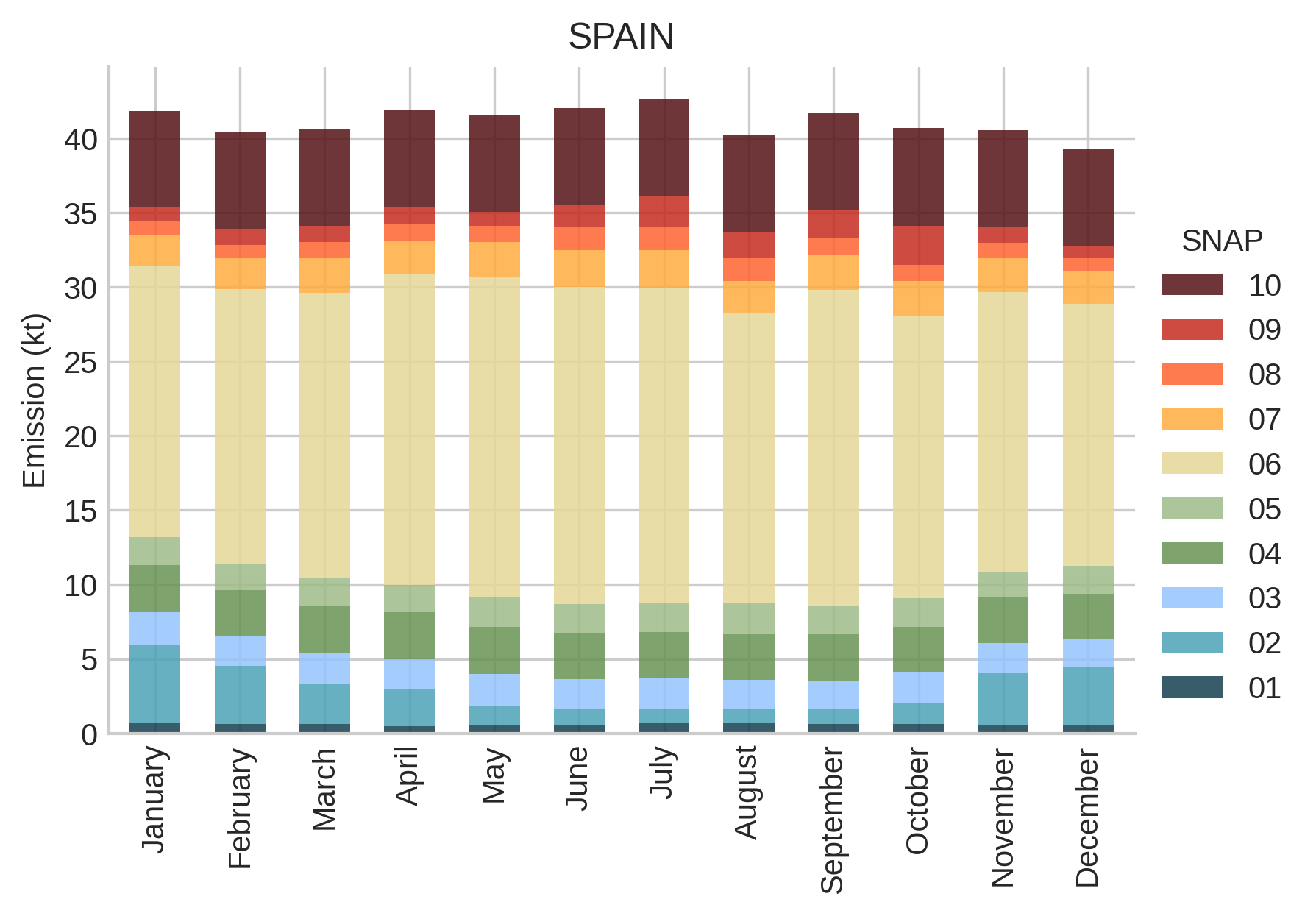 | 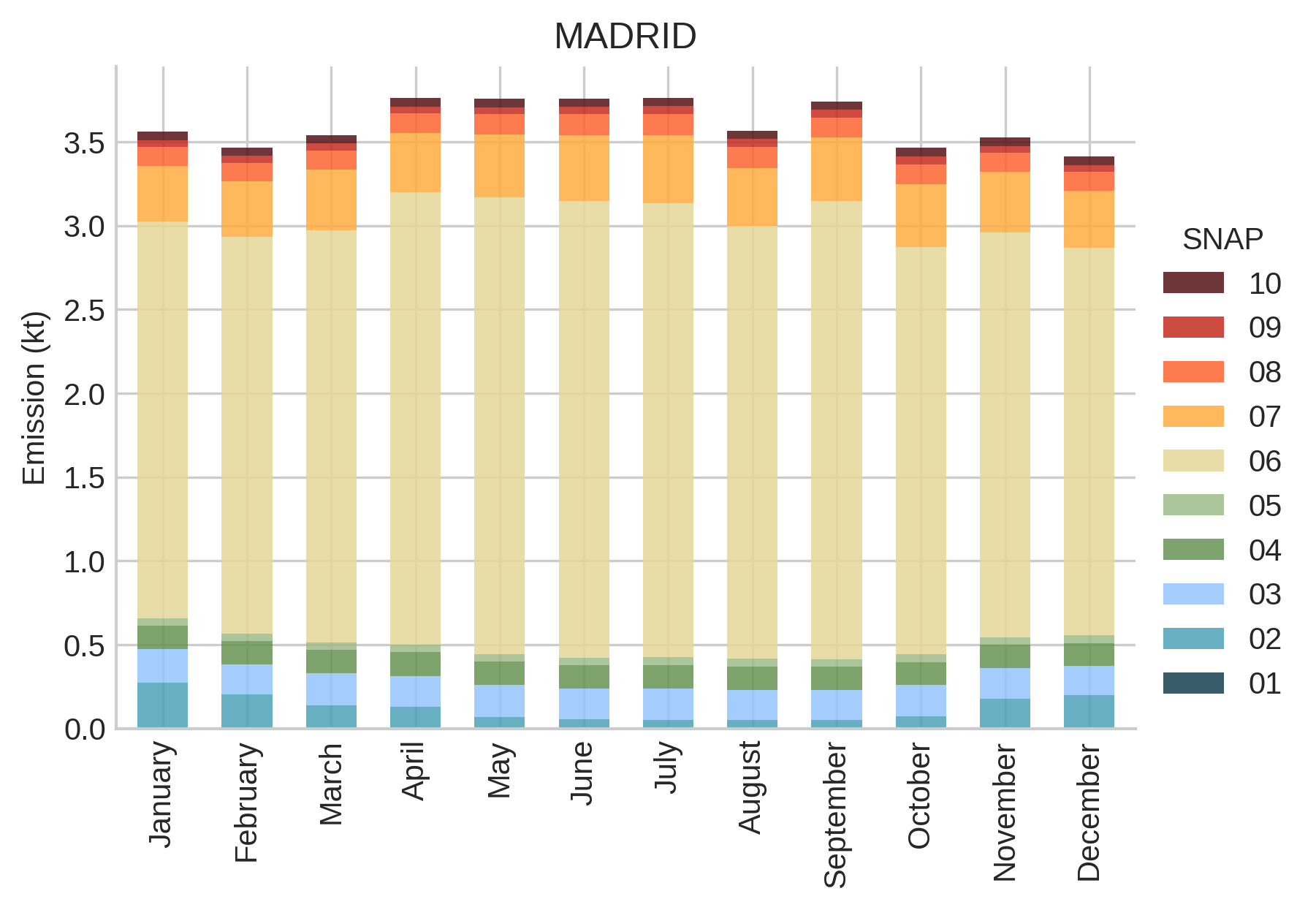 |
| --- | --- |
| 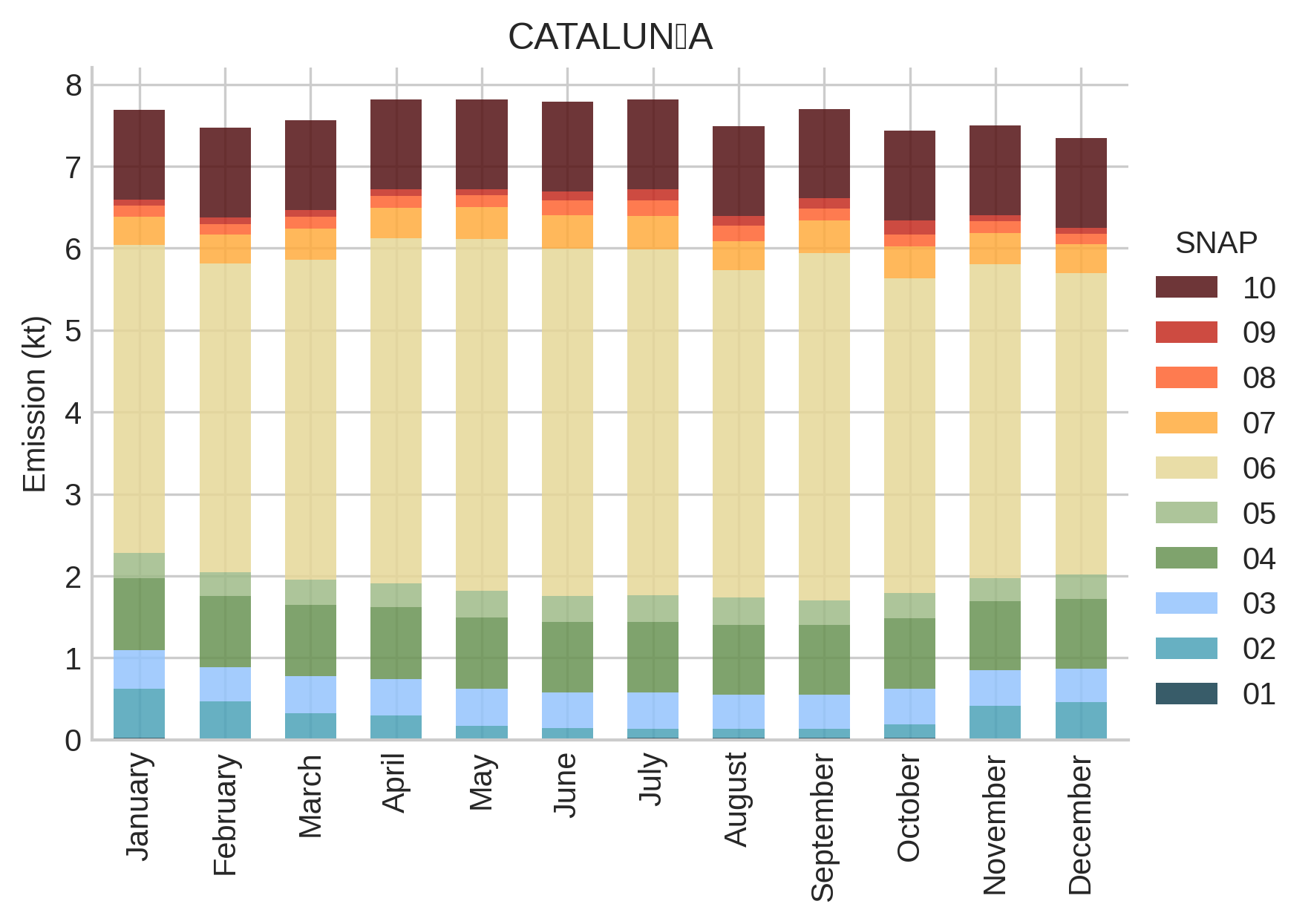 | 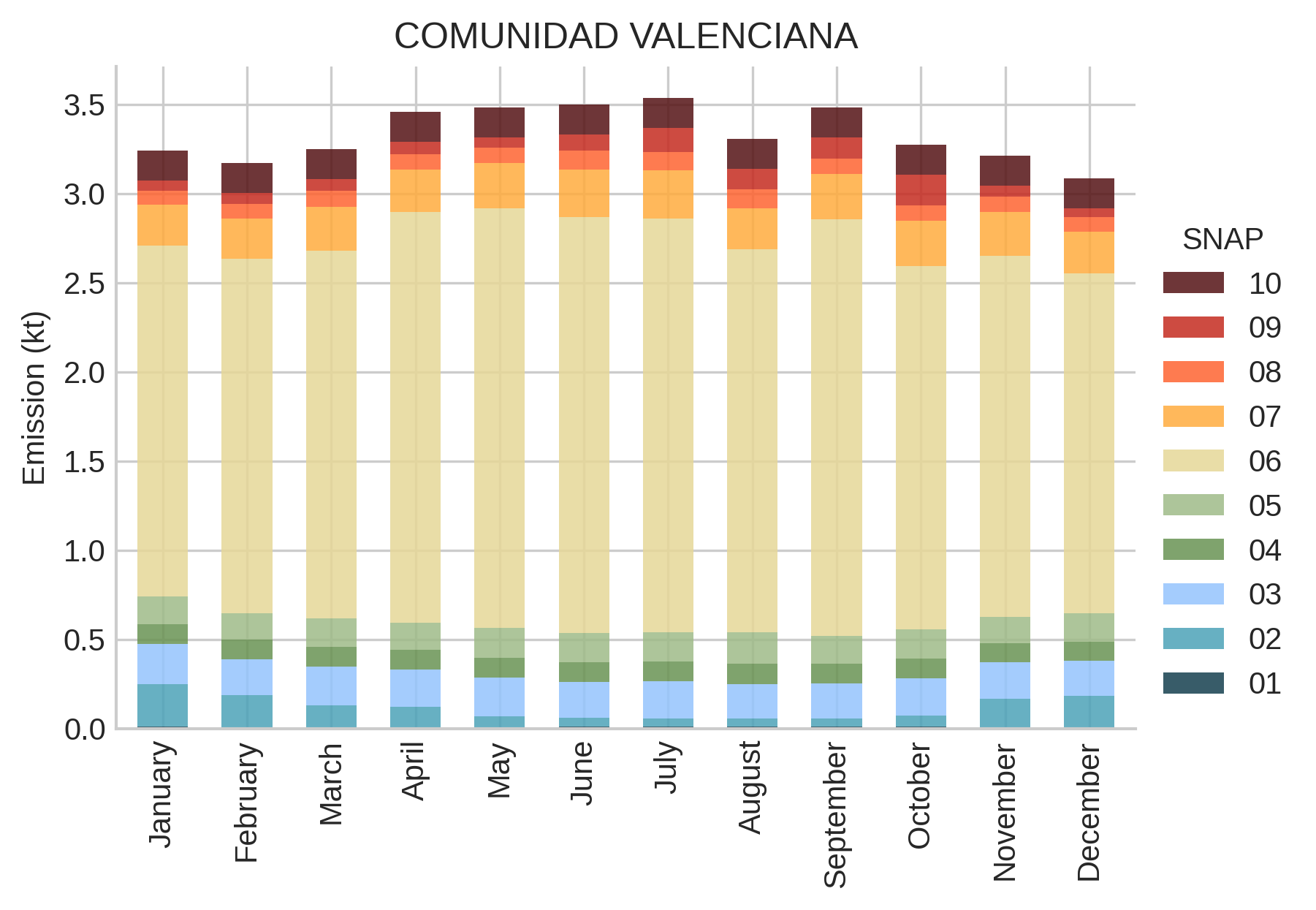 |
| 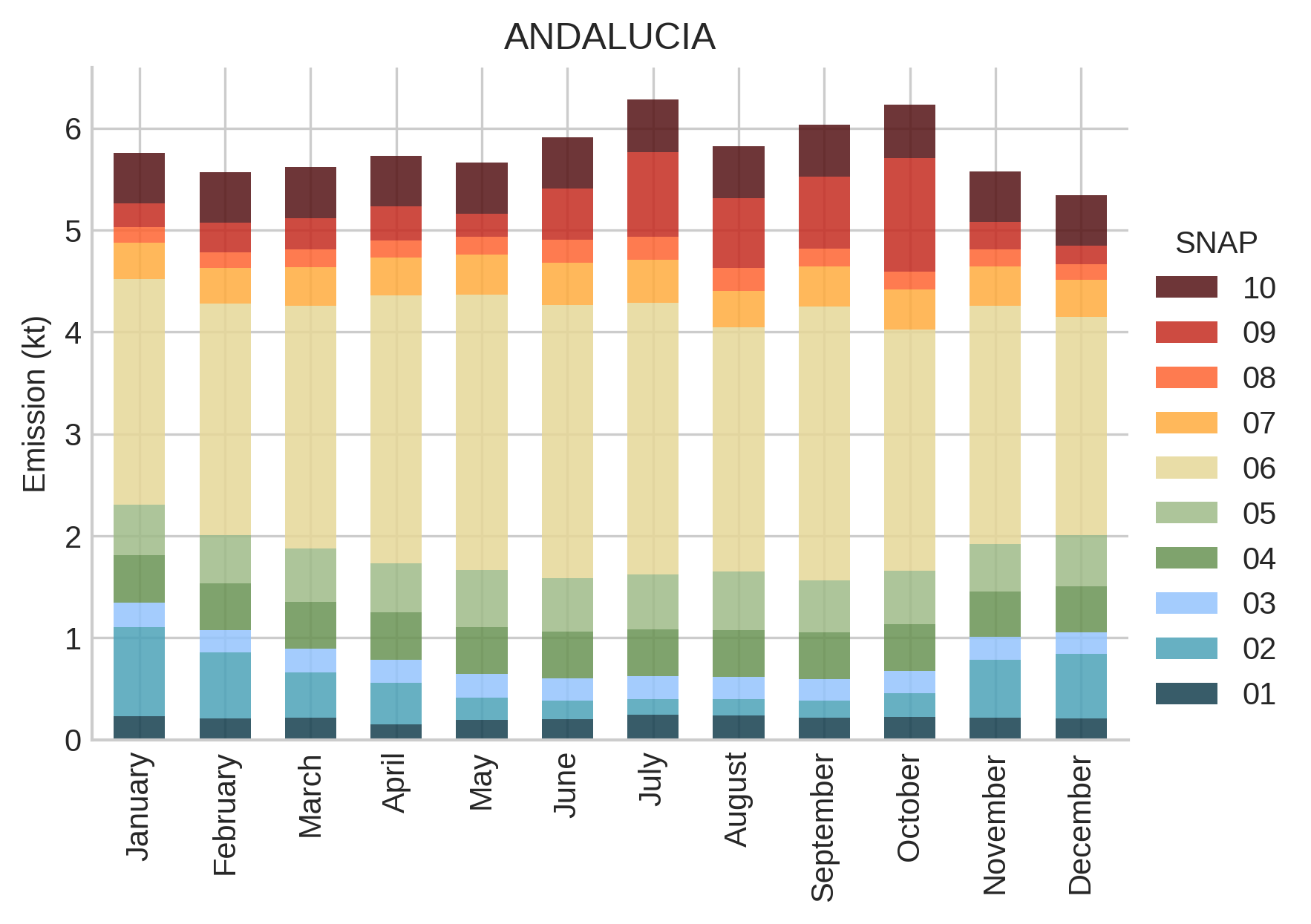 | 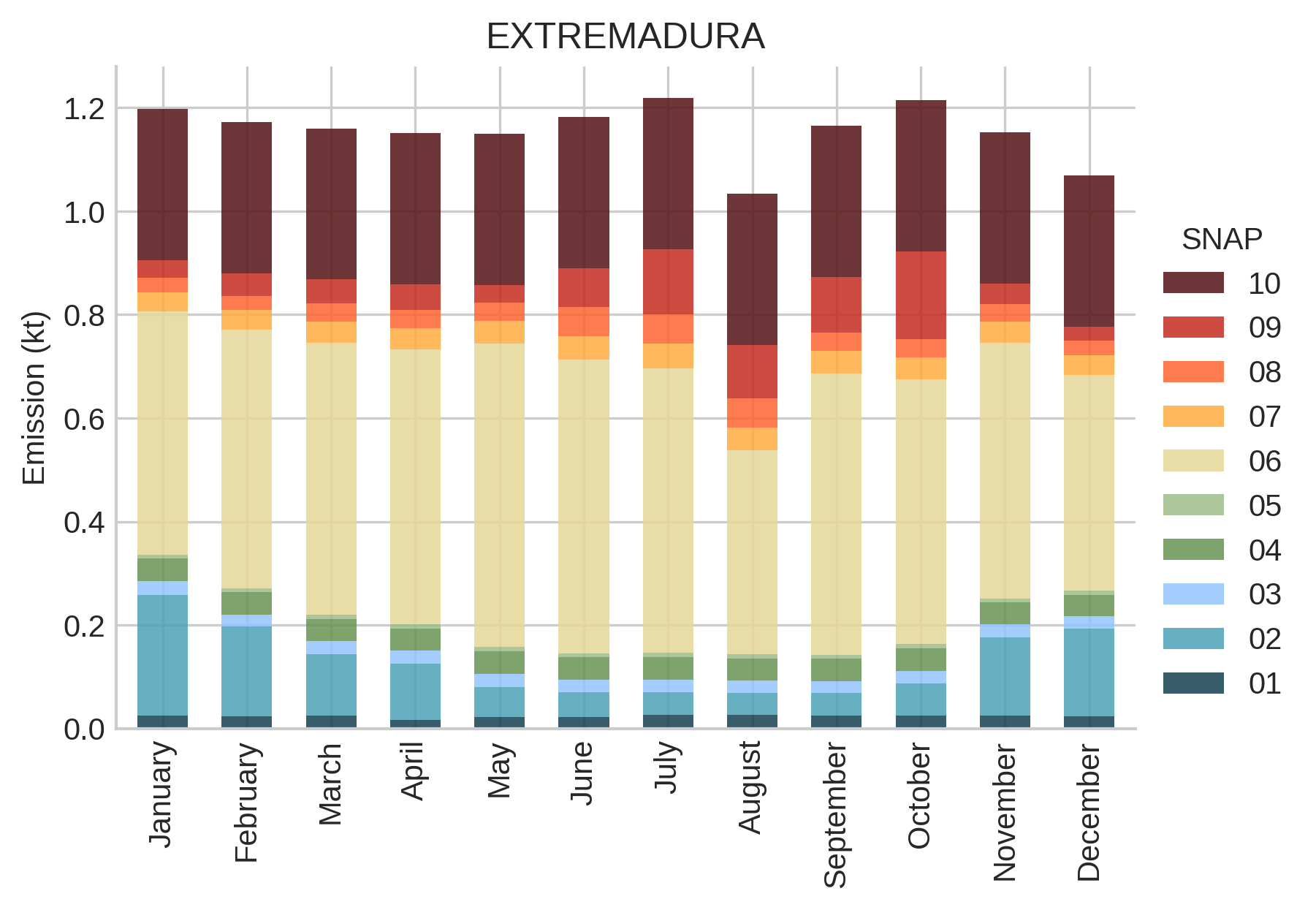 |

Figure S2: Monthly variation of sector contribution to total NMVOC emissions (kt) for Spain and selected regions for 2019.
